# Partially RepRapable automated open source bag valve mask-based ventilator

**DOI:** 10.1016/j.ohx.2020.e00131

**Published:** 2020-08-11

**Authors:** Aliaksei Petsiuk, Nagendra G. Tanikella, Samantha Dertinger, Adam Pringle, Shane Oberloier, Joshua M. Pearce

**Affiliations:** a Department of Electrical & Computer Engineering, Michigan Technological University, USA; b Department of Materials Science & Engineering, Michigan Technological University, USA; c Biomedical Engineering, Michigan Technological University, USA; d Équipe de Recherche sur les Processus Innovatifs (ERPI) , Université de Lorraine, France; e School of Electrical Engineering, Aalto University, Finland

**Keywords:** Ventilator, Pandemic, Ventilation, Influenza pandemic, Coronavirus, Coronavirus pandemic, Pandemic ventilator, Single-limb, Open source, Open hardware, COVID-19, Medical hardware, RepRap, 3-D printing, Open source medical hardware, Embedded systems, Real-time operating system

## Abstract

This study describes the development of a simple and easy-to-build portable automated bag valve mask (BVM) compression system, which, during acute shortages and supply chain disruptions can serve as a temporary emergency ventilator. The resuscitation system is based on the Arduino controller with a real-time operating system installed on a largely RepRap 3-D printable parametric component-based structure. The cost of the materials for the system is under $170, which makes it affordable for replication by makers around the world. The device provides a controlled breathing mode with tidal volumes from 100 to 800 mL, breathing rates from 5 to 40 breaths/minute, and inspiratory-to-expiratory ratio from 1:1 to 1:4. The system is designed for reliability and scalability of measurement circuits through the use of the serial peripheral interface and has the ability to connect additional hardware due to the object-oriented algorithmic approach. Experimental results after testing on an artificial lung for peak inspiratory pressure (PIP), respiratory rate (RR), positive end-expiratory pressure (PEEP), tidal volume, proximal pressure, and lung pressure demonstrate repeatability and accuracy exceeding human capabilities in BVM-based manual ventilation. Future work is necessary to further develop and test the system to make it acceptable for deployment outside of emergencies such as with COVID-19 pandemic in clinical environments, however, the nature of the design is such that desired features are relatively easy to add using protocols and parametric design files provided.

## Graphical abstract

**Figure:**
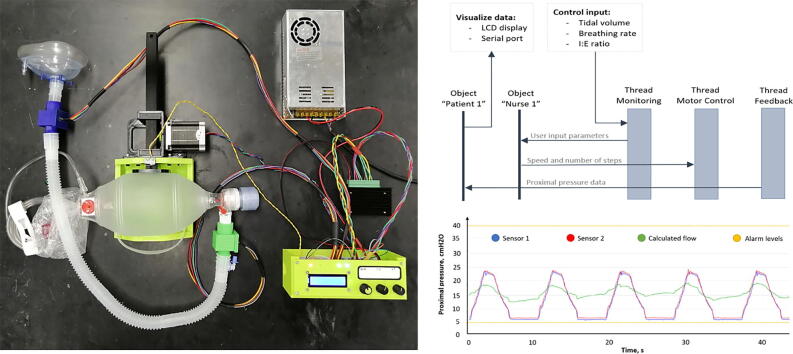

## 1 Specifications table

**Table:** 

Hardware name	RepRapable Automated Open Source BVM-based Ventilator
Subject area	• Medical
Hardware type	• Medical hardware
Open Source License	GNU General Public License (GPL) v3.0 and CERN Open Hardware License (OHL) v1.2
Cost of Hardware	*< $170*
Source File Repository	*https://osf.io/fjdwz/*

## 2 Hardware in context

Coronavirus disease 2019 (COVID-19) is increasing mortality rates by overwhelming medical infrastructure at the regional level [1], [2], [3], [4]. Mechanical ventilators, which are essential for treating both influenza and COVID-19 patients in severe acute respiratory failure [5], [6], are in critical short supply in some locations [7], [8], [9], [10]. During pandemics intensive care units (ICUs) do not have sufficient ventilators to treat all the patients requiring them [11], [12], [13], which forces triage and rationing [14], [15]. This is despite national stockpiles of proprietary, mass-manufactured ventilators, which are simply not numerous enough due to prohibitive costs to service society during an extreme pandemic [16], [17], [18], [19], [20].

Another approach to provide products uses the technically and economically-viable open source small-scale digital technologies and off-the-shelf components for distributed manufacturing [21], [22]. There has already been a concerted effort to apply open source hardware and 3-D printing during the COVID-19 pandemic [23], [24], [25], [26], [27], [28], [29]. In addition, challenges with supply chains during any type of pandemic can be partially offset by open source recyclebots [30], [31], [32], [33], [34] and direct recycling extrusion [35] to close the loop on material supplies with local waste converted into additive manufacturing feedstock [36], [37], [38], [39], [40], [41]. The distributed manufacturing of scientific equipment has been shown to provide custom, high-quality scientific tools for substantially lower costs than conventional proprietary products [42], [43], [44], [45], [46]. This is because a scientific tool can be developed once and then digitally replicated for approximately the cost of the materials [47] creating enormous distributed value [48] a high return on investment [49], and the ability to focus investments for strategic national goals [50], [51]. This same open source hardware design approach [52] can be applied to medical equipment [53], [54], [55], [56] to overcome supply shortages [57], [58], [59], [60], [61].

There has already been some effort in developing low-cost ventilators in the literature [62], [63], [64], [65], [66], [67], [68], [69], [70] as well as in the maker community; however, the former failed to provide full source code and the latter (as of March 2020) was unvalidated and largely untested [71]. To both fill the current critical need for ventilators as well as provide a basis for future pandemics, this article provides the full source code for a fully-functional low-cost 3-D printable open-source pandemic ventilator and includes validation testing using an artificial lung.

## 3 Hardware description.

**Fig. 1:**
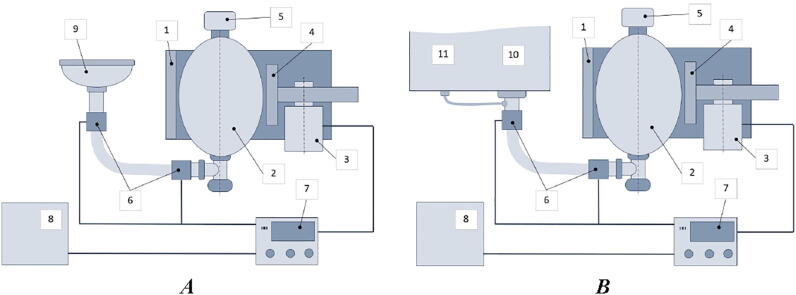
RepRapable Ventilator System: A) standalone automated BVM-based resuscitation system, B) testing procedure, 1) bag mounting system, 2) self-inflating bag, 3) motor setup, 4) compression mechanism (pusher), 5) Positive End Expiratory Pressure (PEEP) valve [85], 6) feedback pressure sensors, 7) control system, 8) power supply with backup battery, 9) air mask, 10) mechanical lung, 11) airway pressure sensor.

The open-source pandemic single-limb (with one hose for the respiratory circuit; exhalation occurs through a single orifice located at the distal end of the circuit) ventilator (Fig. 1) was designed to be highly reproducible, simple in fabrication, maintenance and use for epidemics, pandemics and in developing and under-resourced communities. The design of the device and software was governed by ISO standards [72], the British Medicines & Healthcare Products Regulatory Agency’s rapidly manufactured ventilator system [73] and the Key Ventilation Specifications developed by the E-Vent project [74] along with consultation with health care professionals. The system can be fabricated from readily accessible components, open source Arduino microcontrollers [75], [76] open source electronics that can be made with open source mills [77], [78], [79] and custom parts with a RepRap-class material extrusion-based 3-D printer [80], [81], [82], [83], [84]. Mechanical ventilation, which can be easily controlled by a simple user input, was chosen to be most effective at treating the largest number of people.

The system implements two following modes: controlled mechanical ventilation (CMV) and inverse ratio ventilation (IRV). A user can control breathing rates (breaths per minute or BPM), tidal volume (V_T_, air volume pushed into the lungs), inspiratory/expiration time ratio (I/E ratio). All the mechanical components (Fig. 1: components 1, 4, 7) were developed in open-source CAD systems. The use of a parametric OpenSCAD generator of 3-D printable components (junction boxes for the feedback pressure sensors (Fig. 1) allows to fit any tubing system. A backup battery enables short-term patient mobility and safety protocols in software provide alarm signals when the monitored proximal pressure exceeds the permissible range, or the pressure sensors are disconnected.

**Fig. 2:**
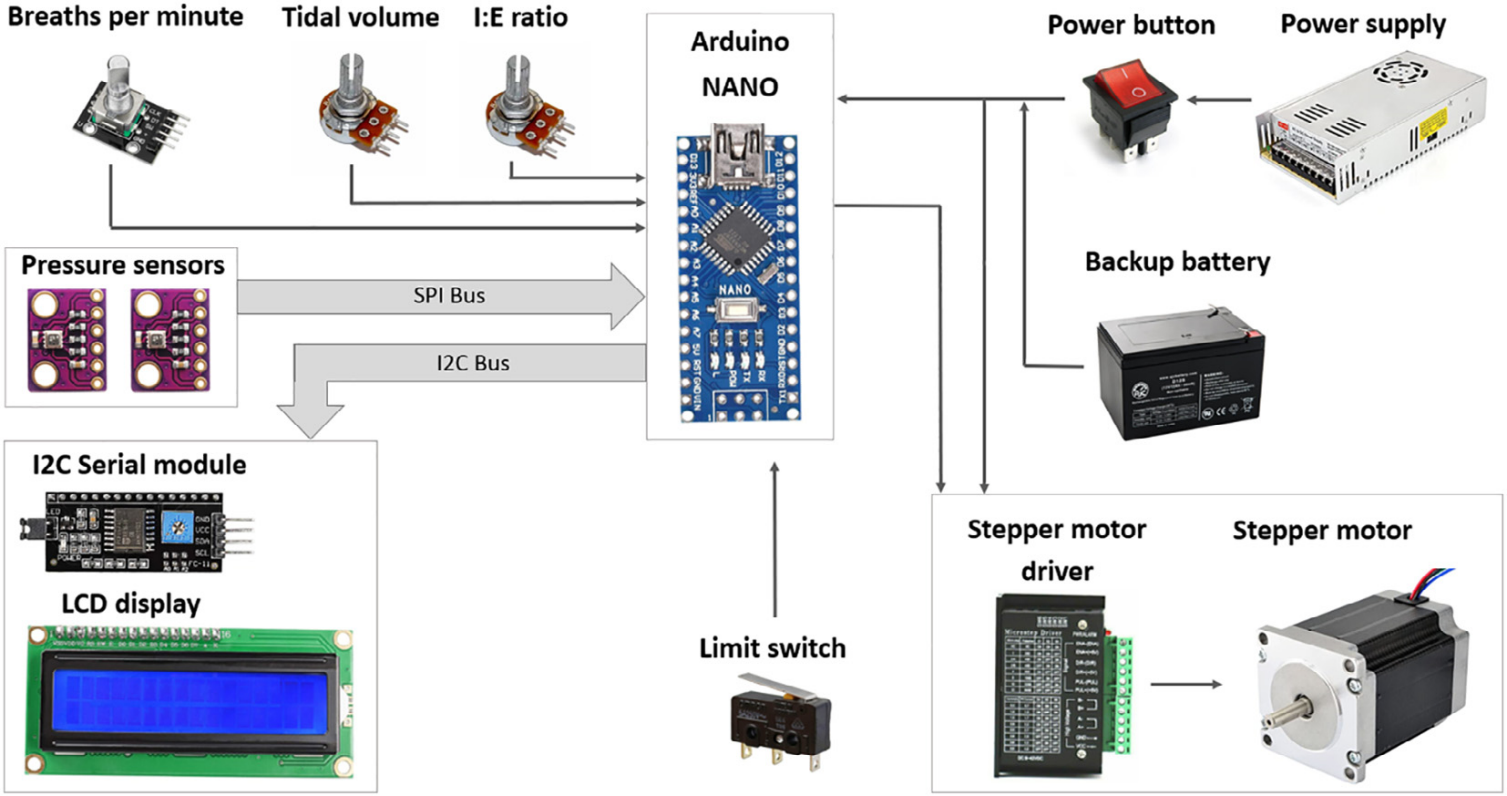
Electrical system architecture.

The electrical architecture is illustrated in Fig. 2. The development process of a medical device as an embedded real-time system can be divided into the main following steps:

1. System design
2. Schematic development
3. Fabrication and assembly
4. Software development
5. Testing

Each of the above steps undergoes numerous iterations, starting with a concept passing the basic and detailed engineering stages, and ending with a finished product [86], [87], [88], [89], [90], [91].

This study of ventilator systems is based on fundamental works [92], [93], [94], [95], [96], [97]. In addition to the technical difficulties with the development of an embedded real-time system, there are also a significant number of details associated with the fabrication of parts that are used in contact with the patient.

The developed system has three control inputs for the variables: tidal volume (V_T_), breathing rate per minute (BPM), and inspiratory-to-expiratory ratio (I/E). BPM and I/E are controlled by rotary potentiometers, and BPM is controlled with a rotary encoder. Having a rotary encoder with an additional button may allow developers to upgrade the system in the future (for example, add a menu to select another mode).

**Fig. 3:**
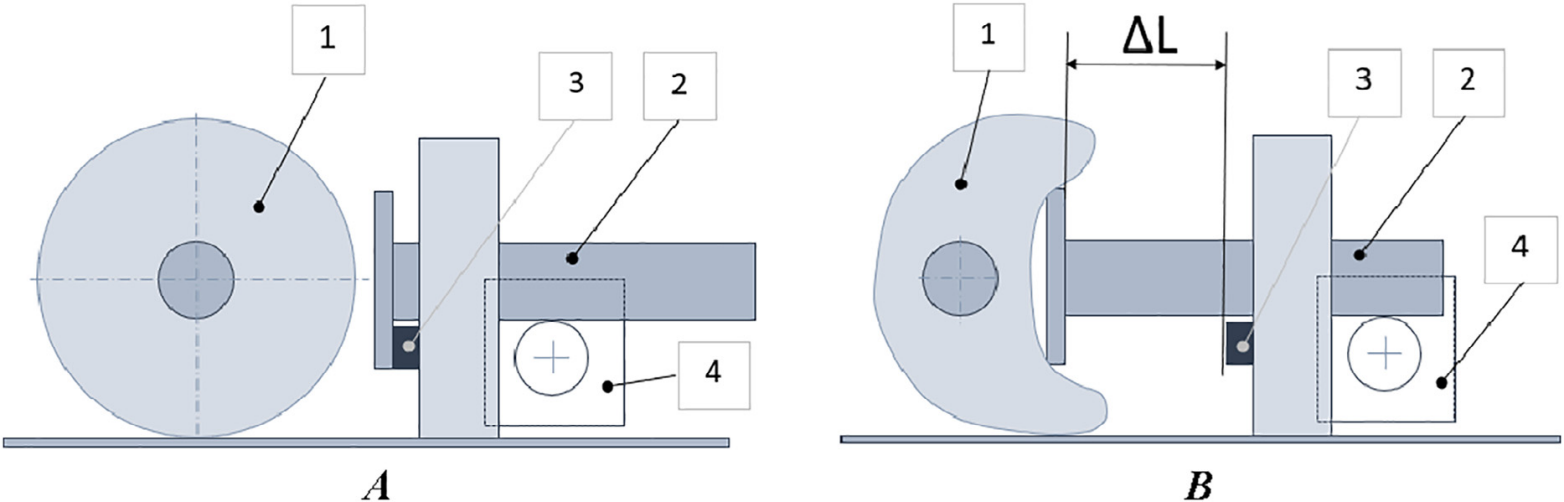
**The** process of compression of a self-inflating bag: A) initial homing position of the pusher, B) compression stage, 1) self-inflating bag, 2) pushing rod, 3) limit switch, and 4) stepper motor.

The self-inflating bag compression process is shown in Fig. 3. At the beginning of the operation, the pusher reaches the home position by hitting the limit switch. From this point, the tidal volume can be adjusted by the amplitude of the movement of the pusher (ΔL), and the breathing rate can be adjusted by a pusher frequency.

**Fig. 4:**
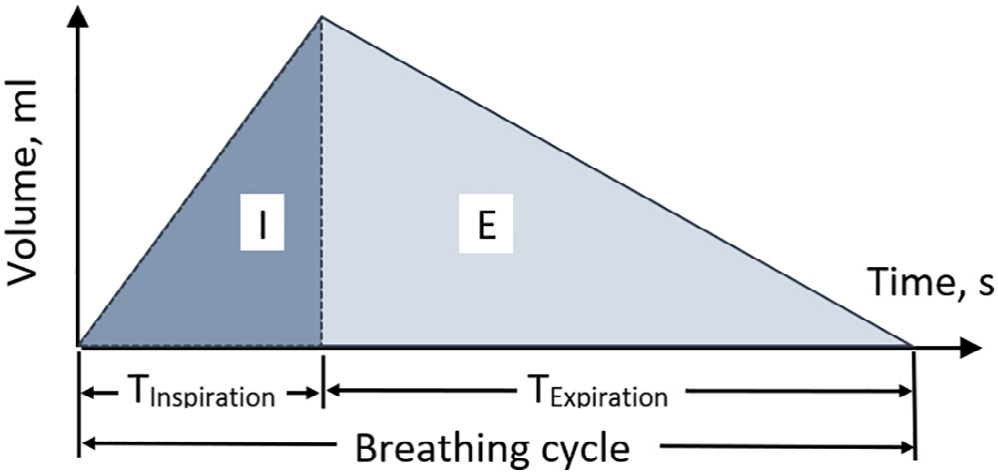
Breathing control diagram: the tidal volume depends on the length of extension of the pusher, and the timings for the inspiratory and expiratory phases – are functions of stepper motor delays between its successive steps.

A breathing control diagram is presented in Fig. 4. According to the stepper motors datasheets [98], [99], both the widely used NEMA-17 and NEMA-23 stepper motors have 1.8 degrees per step, which would give N = 365/1.8 ≈ 203 steps per one revolution of the shaft. With specified micro-stepping multiplier, k = 2…16, it is possible to increase the number of steps per one revolution and provide a more smooth and stable rotation of the motor shaft.

The thrust of the motor depends on the motor torque and the diameter of the gear according to the following equation: F = 2 T/R, where R – is the gear radius and T – is the motor torque. Therefore, by varying the motor current and the size of the gear, it was experimentally found that the herringbone gear (double helical gear) with a diameter of 15 mm will provide reasonable thrust and consistency of contacts between the gear teeth.

As can be seen from Fig. 4, V_T_, BPM, and I/E are functions of the number of steps and the speed of the stepper motor. To provide the desired breathing parameters, the number of motor steps should be calculated as follows:

(1) $$n=\frac{\Delta L∙N}{\pi ∙D}$$

where D is the gear diameter in millimeters, ΔL is the desired pusher length in millimeters, and N is the number of steps per one full revolution. At the same time, N = k ∙ 365/1.8 steps, where k is the micro-stepping multiplier (usually k varies from 2 to 16).

A greater number of steps per revolution of the motor shaft allows smooth rotation and prevents unwanted vibration of the entire apparatus. It is worth noting, however, that the use of micro-stepping higher k values reduces the overall torque of the motor. Thus, a balance was experimentally found between the number of motor steps and the permissible vibration of the bag support system with a micro-stepping coefficient of 4, which corresponds to ~800 steps per single revolution of the shaft.

**Fig. 5:**
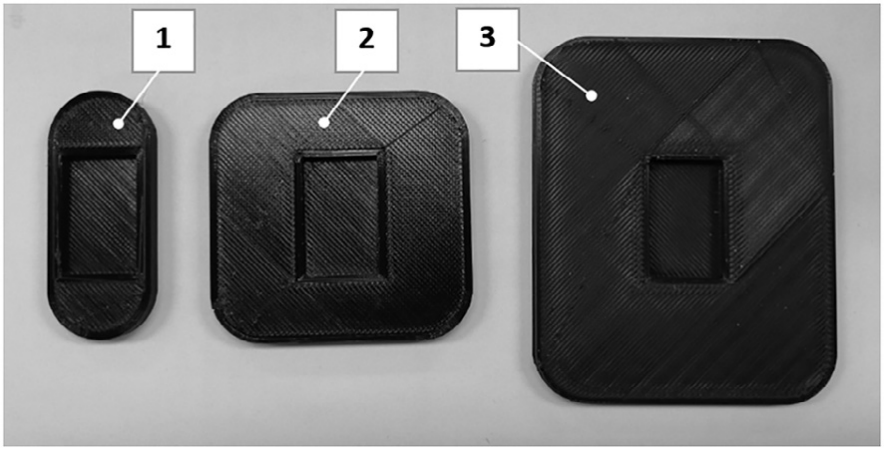
Pushers used for the experiments: 1) 14 cm^2^, 2) 42 cm^2^, 3) 74 cm^2^. The recess shown is the press-fit attachment point for the rack printed part.

**Fig. 6:**
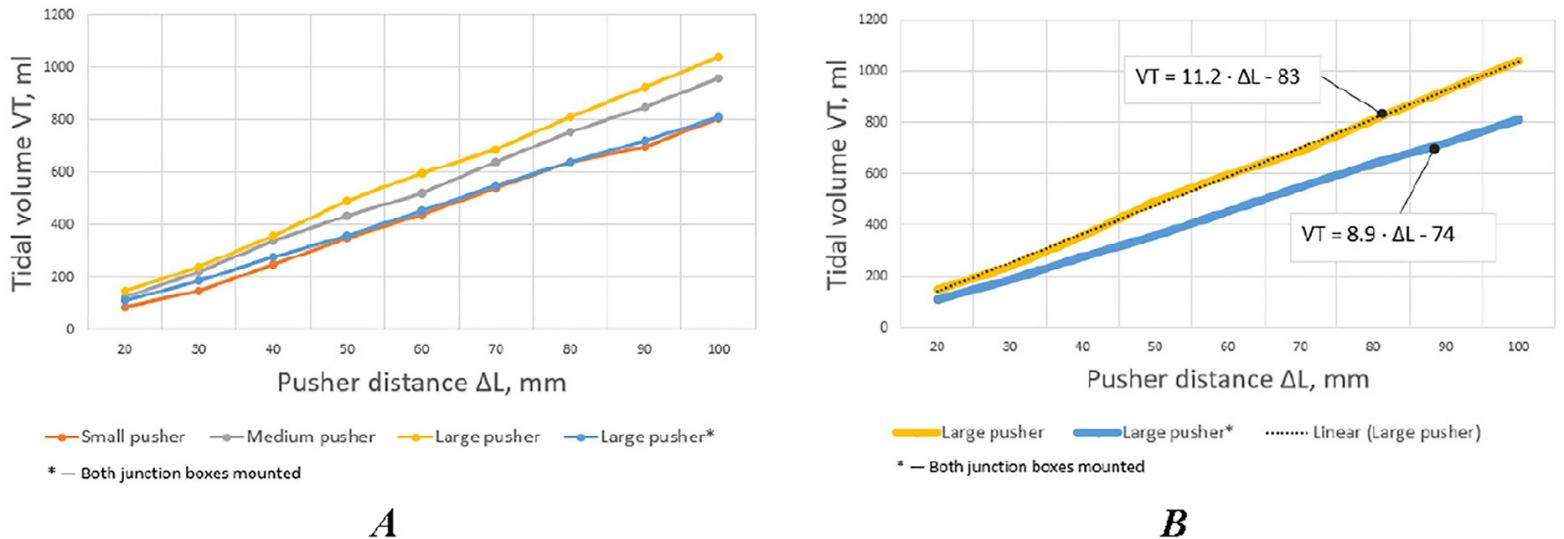
Correspondence of tidal volume and pusher travel distance: A) pushing rod with different pushers and the whole system is without feedback pressure sensors (junction boxes dismounted), B) pushing rod with the large pusher with (*) and without junction boxes mounted.

The volume of air or gas mixture provided by the self-inflating bag is largely due to the shape and size of the pusher. The experiments with three pushers with a total area of 14, 42, and 74 square centimeters (Fig. 5) revealed linear relationships between the volume of air supplied to the lungs and the pusher travel distance (Fig. 6, A). The linear dependency between the pusher travel distance and provided tidal volume equals to ΔL = (83 + V_T_)/11.2 mm.

It should also be noted that air leakage [100], [101] due to the mounting design of the pressure sensors can lead to a decrease in the angle of inclination of the calibration curve (Fig. 6, B).

Thus, the number of motor steps to push the plunger in order to provide the desired air volume can be expressed as follows:

(2) $$n=\frac{\left(V_{T}+83\right)∙365∙k}{8.9∙1.8∙\pi ∙D}$$

where V_T_ is the tidal volume in milliliters, k is the micro-stepping multiplier and D is the gear diameter in millimeters.

Manipulating the BPM and IE control knobs (Fig. 2), it is possible to set the specified breathing parameters by adjusting the time delays between successive motor steps:

(3) $$\Delta_{\mathrm{ti}}=\frac{60}{n∙BPM∙\left(1+\frac{1}{I/E}\right)}$$

where Δti is the time delays (in seconds) during the inspiratory phase of the breathing cycle, BPM is the breathing rate (breaths per minute), I/E is the inspiratory-to-expiratory ratio. The time delays for the expiratory phase will be equal to Δte = Δti∙(I/E)^−1 s^.

Two pressure sensors located at the edges of the air duct are used to calculate proximal airflow using the simplified Bernoulli equation (4) [102], [103]. Sensitive elements of pressure sensors are based on piezo-resistive technology [104], which ensures accuracy, linearity, and stability during long-term operation. Healthcare devices and applications represent the typical use of the given devices. The value of the airflow is not used as a feedback signal and is meant for illustrative purposes only.

(4) $$Q=m∙\sqrt{\Delta p}$$

where Q is the flow rate in liters per minute, Δp is the pressure difference (pressure drop) between two sensor readings in pascals, and m is the calibrated scaling factor.

**Fig. 7:**
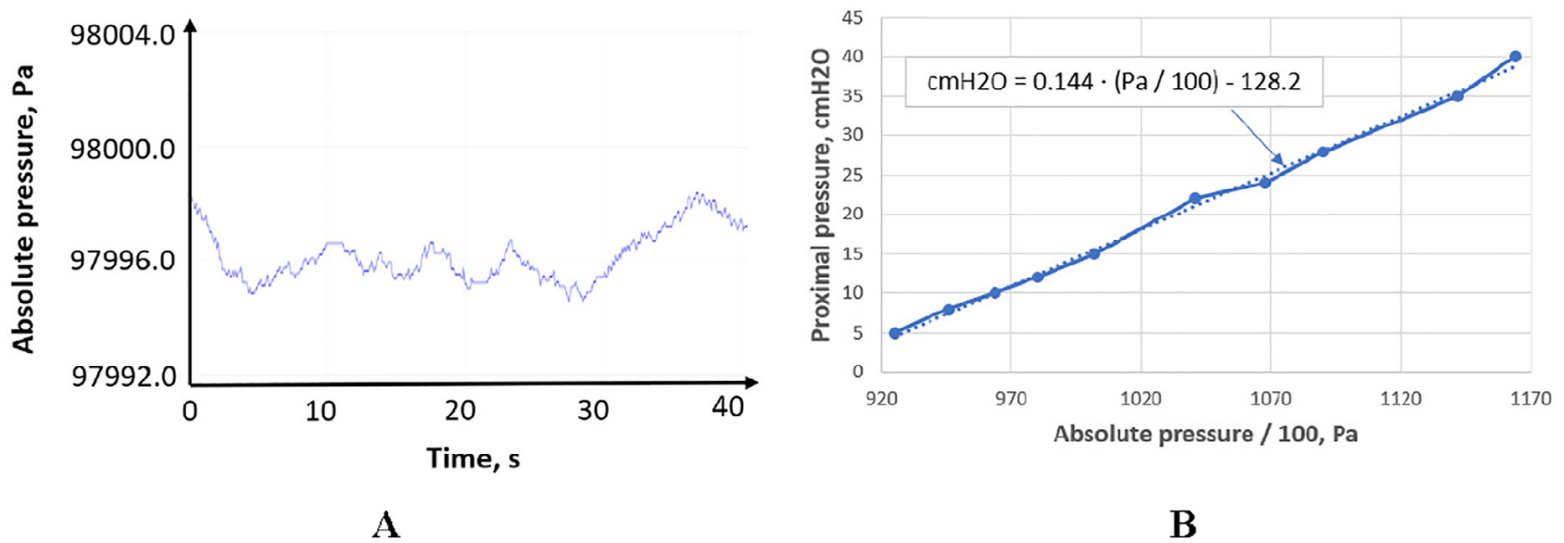
Proximal pressure calibration**:** A) absolute pressure of the laboratory environment (zero-level for proximal pressure), B) BMP280 calibration curve for proximal pressure.

The BMP280 sensor measures the absolute pressure in the range of 300 to 1100 hPa. Therefore, it is necessary to calibrate the system each time the ventilator is used to determine the level of normal ambient pressure (Fig. 7). For these purposes, an additional sensor can also be used to isolate atmospheric pressure so that a pair of BMP280 sensing elements will allow measuring the relative proximal pressure in the airways.

To suppress the noise of the signal from the pressure sensors, an exponential filter is used [105]. This smooths the curve without using significant memory resources. When a new measured value p_t_ is provided, the exponential filter updates a smoothed observation, S_t_:

(5) $$S_{t}=\alpha ∙p_{t}+\left(1-\alpha \right)∙S_{t-1}$$

where S_t-1_ is the previous output value of the filter in pascals, p_t_ is the new measured value in pascals, and α is the smoothing constant (0<α<1).

Since the BMP80 pressure sensors are located in the junction boxes (Fig. 1), and not directly in the airflow path, their readings must be brought to real proximal pressure values based on the results of experiments with the mechanical lung [106].

A calibration curve coerces the sensors values to proximal airway pressure can be described by the following equation:

(6) $$P_{\mathrm{proximal}}=0.144∙\left(\frac{P_{\mathrm{absolute}}}{100}-128.2\right)$$

where P_proximal_ – proximal pressure in cmH_2_O, P_absolute_ – absolute BMP280 pressure in pascals.

**Fig. 8:**
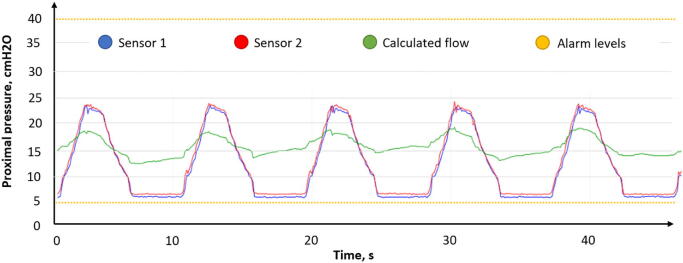
Pressure sensors feedback.

Thus, the signals from pressure sensors located at opposite ends of the airway can be interpreted as proximal pressure. Based on the above Eq. (6), it is possible to determine the readings of the sensors corresponding to the minimum allowable PEEP pressure and the maximum critical pressure of 40 cmH_2_O (Fig. 8).

The control system is based on the Arduino controller and a stepper motor setup (NEMA-23 motor). The Arduino Nano board was chosen as a controller due to low relative expense while having sufficient digital and analog pins.

A significant number of medical software development standards contain information and requirements regarding software design, validation, and certification [107], [108], [109], [110], [111], [112]. However, in the global pandemic, meeting all requirements can be difficult. The main guidelines for emergency ventilation systems is the use of real-time operating systems and a serial peripheral interface for connecting sensing devices [113].

The use of an open-source real-time operating system (FreeRTOS) library [114] for Arduino considerably expands the possibilities of the controller. A real-time operating system provides essential functions to software tasks, such as scheduling, dispatching, inter-task communication, and synchronization [115].

**Fig. 9:**
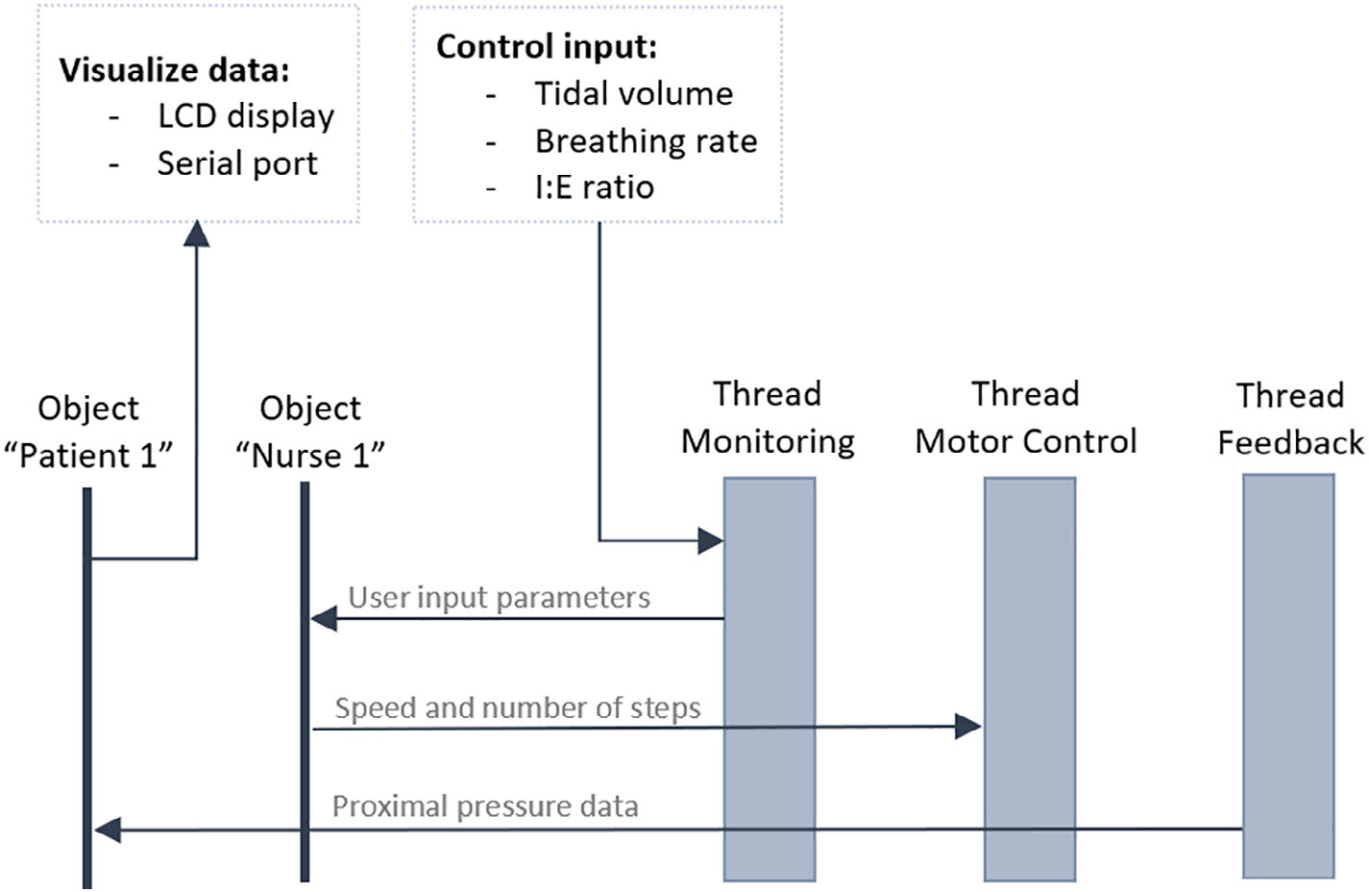
Software system architecture.

The software system architecture is shown in Fig. 9. There are three parallel tasks with equal priorities communicating with the two instances of the patient and nurse classes, which provide scalability (there may be more “patients” and “nurses”, as well as threads with other functions) and possibility of transition to another hardware background since FreeRTOS supports most popular processors and microcontrollers [114].

**Fig. 10:**
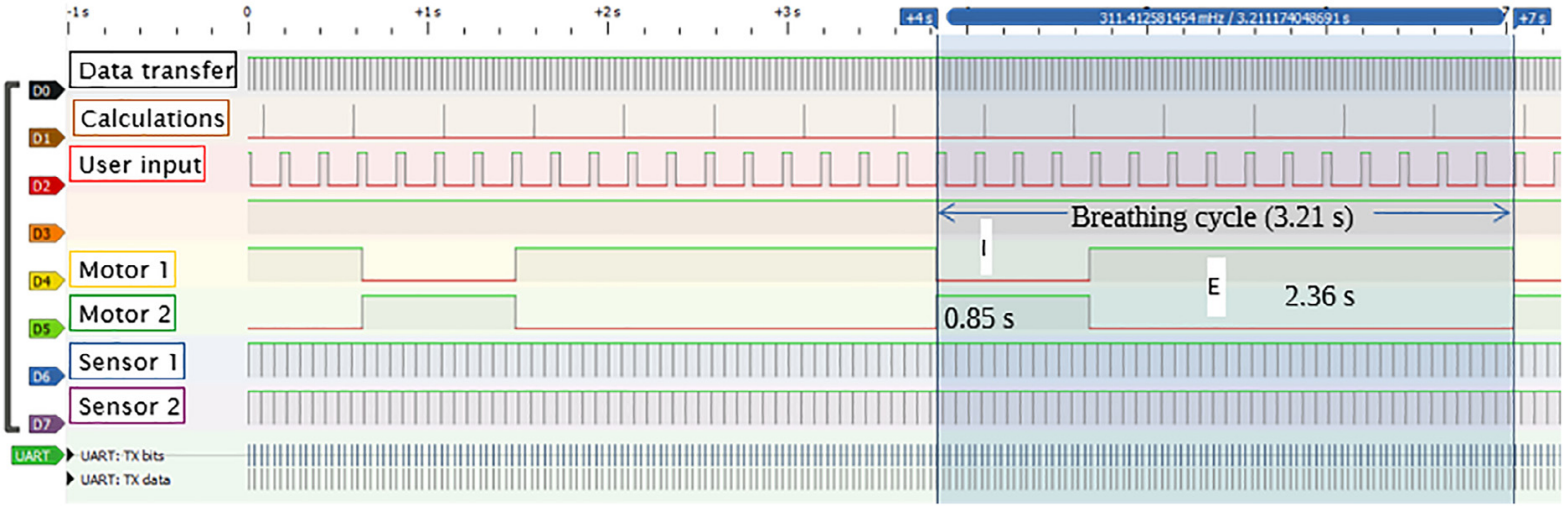
Algorithm trace: example for 455 mL of tidal volume, 20 breaths per minute, and 1:3 inspiration-to-expiration ratio.

The software trace (Fig. 10) obtained using a logic analyzer can visualize the execution of the algorithm in terms of the frequency and duration of existing tasks. The main utilities are presented in Table 1.

**Table 1:** Software tracing summary.

Utility	Frequency, Hz	Duration, ms
Serial data transfer	14	2.5
Motor parameters recalculation	2	0.1
Reading user input and LCD display update	4.5	50
Two pressure sensors readings	14	1.5

To summarize the main characteristics of the ventilator can be represented as follows:

• Low cost (~$20 for 3-D printed mechanical components, ~$120 for electronic components, and ~$23 for BVM and single-limb ventilator circuit). Note that this is solely the cost of materials.
• Availability of components for assembly and ease of fabrication.
• Providing a controlled breathing mode with the following parameters:
  1. Tidal volume in the range from 100 to 800 mL
  2. Breathing rate in the range from 5 to 40 BPM
  3. Inspiratory-to-expiratory ratio in the range from 1:1 to 1:4
• Software reliability through the use of the real-time operating system
• Reliability and scalability of measurement circuits through the use of the serial peripheral interface (SPI)
• Ability to connect additional hardware due to the object-oriented algorithmic approach

## 4 Design files

### 4.1 Design files summary

**Table:** 

No.	Design file name	Image	File type	Open source license	Location of the file
*Bag mounting system*					
1	BagSupport.FCStd		FreeCAD	GNU GPL v3	https://osf.io/fjdwz/
2	MotorMount.FCStd		FreeCAD	GNU GPL v3	https://osf.io/fjdwz/
3	Rack.FCStd		FreeCAD	GNU GPL v3	https://osf.io/fjdwz/
4	Pinion.FCStd		FreeCAD	GNU GPL v3	https://osf.io/fjdwz/
5	Rod_head_pusher.scad		OpenSCAD	GNU GPL v3	https://osf.io/fjdwz/

*Junction boxes for pressure sensors*					
6	Junction_box_generator.scad		OpenSCAD		https://osf.io/fjdwz/
7	Pressure_sensor_junction_box.stl		STL	GNU GPL v3	https://osf.io/fjdwz/
8	Pressure_sensor_ junction_box_plate.stl		STL	GNU GPL v3	https://osf.io/fjdwz/

*Control box*					
9	Control_box_panel.FCStd		FreeCAD	GNU GPL v3	https://osf.io/fjdwz/
10	BreadBoxBase.FCStd		FreeCAD	GNU GPL v3	https://osf.io/fjdwz/

*Electrical schematics and printed circuit board*					
11	Vent_Controller_Simple.pdf	—	PDF	GNU GPL v3	https://osf.io/fjdwz/
12	Vent_Controller_Simple.pro	—	KiCAD Project	GNU GPL v3	https://osf.io/fjdwz/
13	Vent_Controller_Simple.sch	—	KiCAD Schematic	GNU GPL v3	https://osf.io/fjdwz/
14	Vent_Controller_Simple.kicad_pcb	—	KiCAD PCB File	GNU GPL v3	https://osf.io/fjdwz/
15	/gerbers	—	Gerber	GNU GPL v3	https://osf.io/fjdwz/

*Arduino firmware*					
12	arduino_firmware.ino	—	Arduino sketch	GNU GPL v3	https://osf.io/fjdwz/

Both the FreeCAD and OpenSCAD files were designed to be parametric to allow future developers to replicate this system for different core components (e.g. different sizes of bags).

1. *“Bag support”* provides support for the bag to keep it stabilized in the transverse and longitudinal directions. Major modifications may involve changing the entire geometry to fit a different self-inflating bag. Minor modifications may include changes to the attachment points to the motor mount part or additional support for the bag.
2. *“Motor mount”* provides a mounting point and support for NEMA-23, it also provides a sliding path for the rack. Major modifications may involve changing the geometry for use with a different motor. Minor modifications may involve changing the attachment points to the bag support, changes to the sliders.
3. *“Rack”* and *“Pinion”* use the motor power to compress the bag. Both the FreeCAD source files were created with the “FCGear” add-on that generates gear profiles. Steps are named for ease of use. Major modifications involve changes to the gear (gear specifications are accessible within the file) which requires “FCGear” workbench. Minor modification involves changes to the geometry of the hole for the motor shaft, changes to the nut-trap, as well as tolerance adjustments.
4. *“Rod head pusher”* is attached to the rack and serves both to compress the bag and to close the limit switch during the homing process.
5. *“Junction box generator”* is the master file for rendering the junction box and plate in order to create a press-fit between two tubes with a sensor epoxied inside.
6. *“Pressure sensor junction box”* and *“Pressure sensor junction box plate”* are the current precise geometries for the ventilator design described in this work. Import into a slicer to use.
7. *“Control box panel”* and *“Breadboard box base”* are the parts of the control system housing with user input.
8. *“Schematic”* is a control system wiring diagram that can be implemented using both a breadboard and a printed circuit board.
9. *“Arduino firmware”* is a program that reads user input and implements motor control in accordance with user-defined breathing parameters.

## 5 Bill of materials

The complete Bill of Materials is available in the OSF repository (https://osf.io/ugt3e/).

### 5.1 Breathing system Bill of materials

**Table:** 

Designator	Number	Cost per unit – USD	Total cost – USD	Source of materials
Adult Bag-Valve-Mask Ambu SPUR II	1	15.95	15.95	https://www.heartsmart.com/ambu-adult-spur-ii-adult-bvm-p
Single-limb ventilator circuit	1	6.71	6.71	https://www.saveritemedical.com/products/adult-single-limb-portable-ventilator-circuit?variant=32484935052&gclid=EAIaIQobChMIzsvWlbye6gIVDtbACh2DswWiEAQYAiABEgL0FPD_BwE

### 5.2 Mechanical system Bill of materials

**Table:** 

Designator	Component	Mass in grams	Cost per unit – USD	Total cost – USD	Source of materials	Material type
PLA – 3-D printer filament	BagSupport.stl, MotorMount.stl, Rack.stl, Pinion.stl, BreadBoardBase.stl, BreadBoardCover.stl	700	$25/kg	$17.50	https://us.polymaker.com/product/polylite-pla/	Hard thermoplastic
Ninjaflex – 3-D printer filament	Junction_box and plate STLs	40	$85/kg	$3.40	https://www.fennerdrives.com/product-lines/_/3d/	Flexible polymer

### 5.3 Control system Bill of materials

**Table:** 

Component	Number	Cost per unit – USD	Total cost – USD	Source of materials
Power supply, 12 V	1	18.95	18.95	https://www.amazon.com/eTopxizu-Universal-Regulated-Switching-Computer/dp/B00D7CWSCG
Battery 12 V 7A	1	17.5	17.5	https://www.amazon.com/ExpertPower-EXP1270-Rechargeable-Lead-Battery/dp/B003S1RQ2S
Breadboard	1	7.9	7.9	https://www.amazon.com/BB830-Solderless-Plug-BreadBoard-tie-Points/dp/B0040Z4QN8
Arduino NANO	1	4.3	4.3	https://www.amazon.com/WYPH-ATmega328P-Microcontroller-Development-Not-soldered/dp/B07KCH534K
Stepper motor NEMA23 (1.9 Nm)	1	32	32	https://www.amazon.com/STEPPERONLINE-Stepper-269oz-Length-Router/dp/B077Z5QJCL
Stepper motor driver TB6600	1	10.9	10.9	https://www.amazon.com/TB6600-Stepper-Driver-Controller-tb6600/dp/B07S64MBTR
Rotary potentiometer 10 K	2	1.2	2.4	https://www.amazon.com/Uxcell-a15011600ux0235-Linear-Rotary-Potentiometer/dp/B01DKCUVMQ
Rotary encoder	1	1.8	1.8	https://www.amazon.com/Cylewet-Encoder-15%C3%9716–5-Arduino-CYT1062/dp/B06XQTHDRR/
Limit switch	1	0.6	0.6	https://www.amazon.com/MXRS-Hinge-Momentary-Button-Switch/dp/B07MW2RPJY
Power switch	1	0.5	0.5	https://www.amazon.com/ZUPAYIPA-Solder-Rocker-Switch-Toggle/dp/B01N2U8PK0/
16x2 LCD display	1	9.0	9.0	https://www.amazon.com/SunFounder-Serial-Module-Display-Arduino/dp/B019K5X53O
Pressure sensor BMP280	2	2.8	5.6	https://www.amazon.com/CHENBO-Barometric-Pressure-Precision-Atmospheric/dp/B01N4EHIW6
Buzzer	1	1.5	1.5	https://www.amazon.com/Cylewet-Electronic-Sounder-Continuous-CYT1117/dp/B07QJG46B8
Fan 12 V	1	6.0	6.0	https://www.amazon.com/ANVISION-Bearing-Brushless-Cooling-YDM4010B12/dp/B0711FVD48
Resistor 200 Ohm 0.25 W	1	0.01	0.01	https://www.amazon.com/McIgIcM-resistor-Electronics-resistors-assortment/dp/B06WRQS97C
Resistor 1 k Ohm 0.25 W	4	0.01	0.04	https://www.amazon.com/McIgIcM-resistor-Electronics-resistors-assortment/dp/B06WRQS97C
Resistor 1 k Ohm 5 W	1	0.89	0.89	https://www.amazon.com/uxcell-Tolerance-Resistance-Electronic-Experiments/dp/B07RWRVWYY
Diode 1 N4007	2	0.05	0.1	https://www.amazon.com/McIgIcM-1 N4007-Standard-Through-Rectifier/dp/B071DXGHL7
Capacitor 100uF 50 V	2	0.05	0.1	https://www.amazon.com/JABINCO-100uf-Aluminum-electrolytic-Capacitor/dp/B082TQRDKT
LED	1	0.05	0.05	https://www.amazon.com/Novelty-Place-Transparent-Electronics-Components/dp/B01AKM9ODG
Bolt M4x20	9	0.06	0.54	https://www.amazon.com/Comdox-500pcs-Socket-Assortment-Threaded/dp/B071VBL355/
Bolt M3x20	8	0.06	0.48	https://www.amazon.com/Comdox-500pcs-Socket-Assortment-Threaded/dp/B071VBL355
Nut M4	9	0.06	0.54	https://www.amazon.com/Comdox-500pcs-Socket-Assortment-Threaded/dp/B071VBL355/
Nut M3	8	0.06	0.48	https://www.amazon.com/Comdox-500pcs-Socket-Assortment-Threaded/dp/B071VBL355/

## 6 Build instructions

The installation of the device consists of three stages: 1) bag holder assembly, 2) breathing system assembly, and 3) control system assembly. To print all components, a RepRap-class 3-D printer with a minimum printing area of 230x230x100mm is needed. Fabrication of all parts takes from 25 to 34 h, depending on print settings. Printing material can be polylactic acid (PLA) or glycol modified polyethylene terephthalate (PETG). For junction boxes with pressure sensors, thermoplastic polyurethane (TPU, NinjaFlex in this work) material was chosen to minimize air leakage.

### 6.1 Bag holder assembly

1. Obtain any RepRap-class 3-D printer with build dimension of at least 230 × 230 × 100 mm.

2. Obtain a minimum of 500 g of 3-D printer filament such as PLA or PETG.

3. Download the files from https://osf.io/fjdwz

4. Make modifications to FCStd file if necessary and export STLs or use provided STLs

5. Import the STL file into a slicing software, such as Cura.

6. Use default printing parameters for the material. Change orientation of the model if necessary. Use a layer height of 0.1–0.25 mm, at least 20% infill and a minimum top/bottom thickness of 0.5 mm and wall thickness of 1 mm. No supports are necessary.

**Fig. 11:**
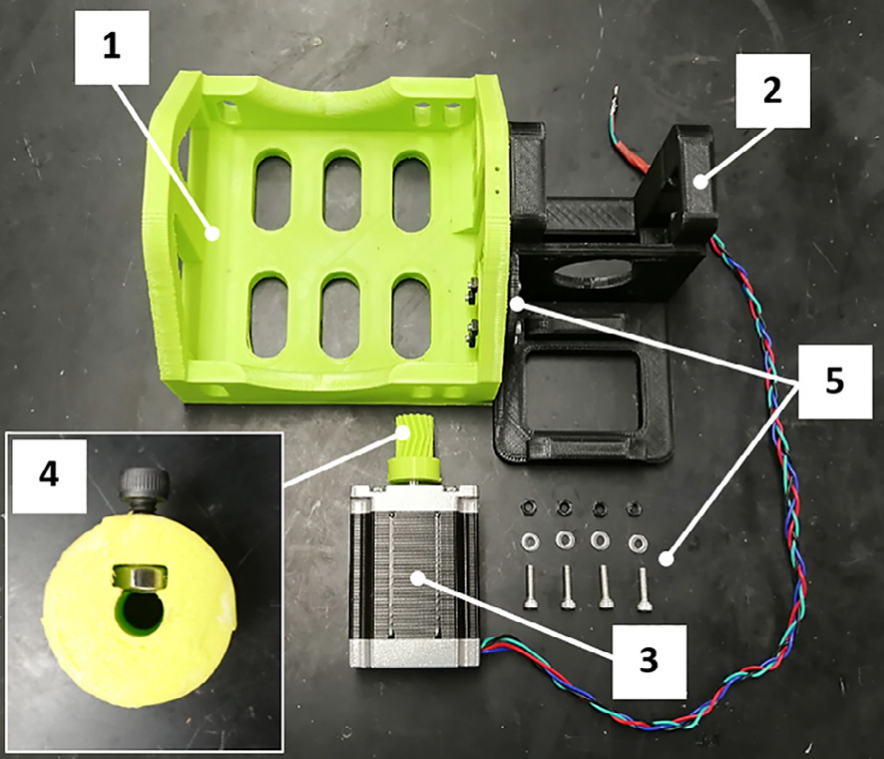
Bag holder assembly: 1) bag support, 2) motor mount, 3) stepper motor, 4) pinion gear, 5) fasteners.

**Fig. 12:**
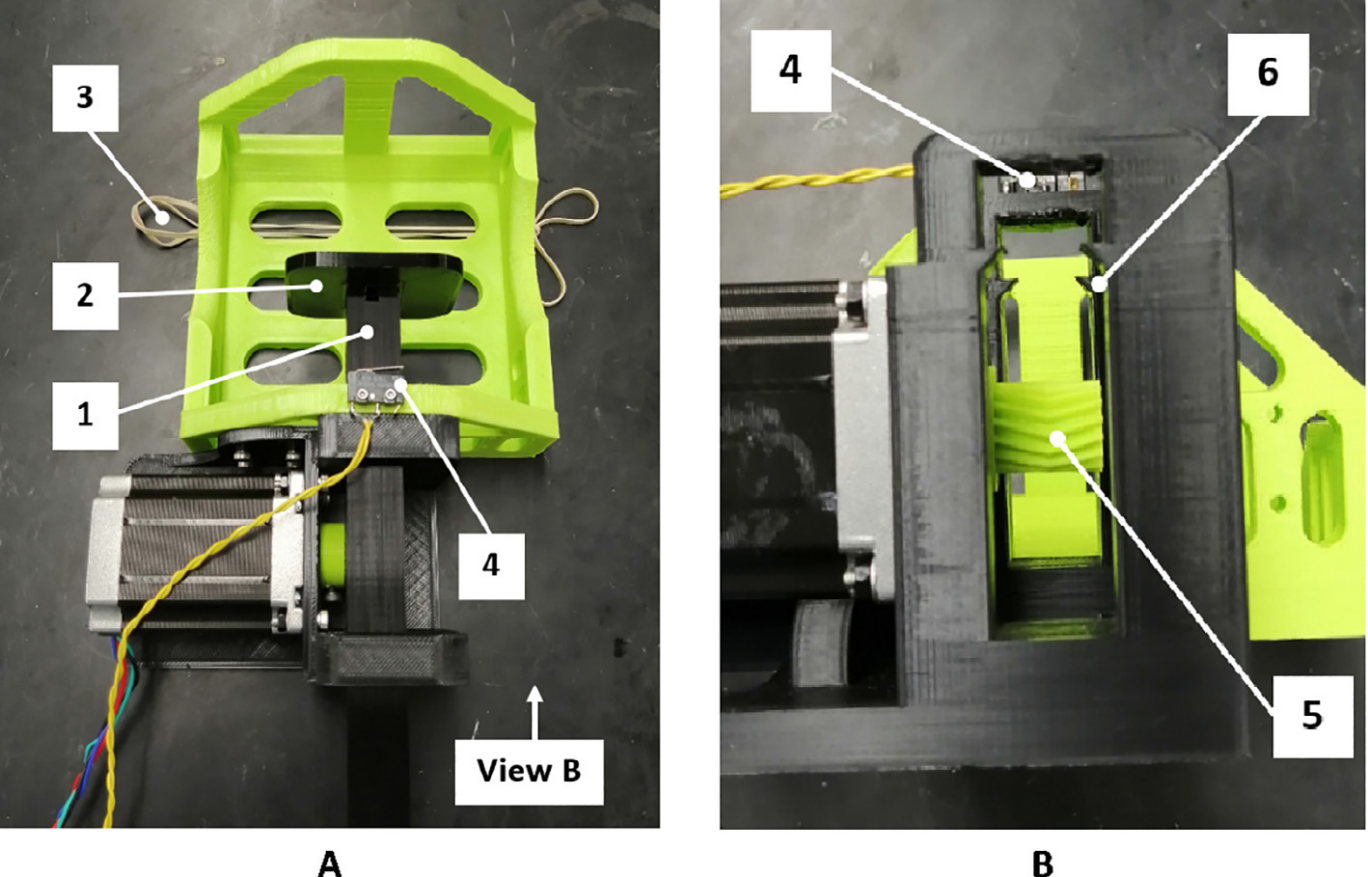
Bag support and motor mount assembly: A) top view, B) view from the side of the pushing rod groove, 1) pushing rod, 2) pusher, 3) rubber band for bag support, 4) limit switch, 5) pinion gear, 6) pushing rod groove.

7 Print time and mass of filament used:

• The print time would be approximately 12–15 h, with around 170–250 g of filament used for bag support.
• The print time would be approximately 7–10 h, with 100–150 g of filament used for motor mount.
• The print time would be approximately 5–7 h, with 70–100 g of filament used for Rack
• The print time would be approximately 1–2 h, with 5–10 g of filament used for Pinion

8. Assemble the parts as shown in Fig. 11. All nine bolts and nuts are M4. It can be modified in the FreeCAD files if necessary.

9. Print and assemble pusher.

• Obtain a common rigid polymer-based filament such as PLA or PETG
• If it is necessary to alter the design Download the Rod_head_pusher.scad from https://osf.io/fjdwz/. Alter the X-Y dimensions and pusher grip length parameters in the design using the variable list as desired.
• If there is no need to alter the design download the Rod_head_pusher.stl from https://osf.io/fjdwz/ and import into Cura.
• Download the material file from https://osf.io/fjdwz/ and import into Cura and set the appropriate print parameters individually or by importing the associated material file from https://osf.io/fjdwz/. 50 min are needed to print the 10 g out of PLA at 100% infill, at 30 mm/s as determined by Cura.
• Once printed as seen in Fig. 12, slot the pusher onto the rod as it is installed in the ventilator. The pusher should press-fit tightly onto the rod.

### 6.2 Breathing system assembly

For fabrication the junction boxes an open source RepRap Lulzbot TAZ 3-D printer was used with Ninjaflex filament and the open source Lulzbot Cura slicer (edition 3.6.8).

**Fig. 13:**
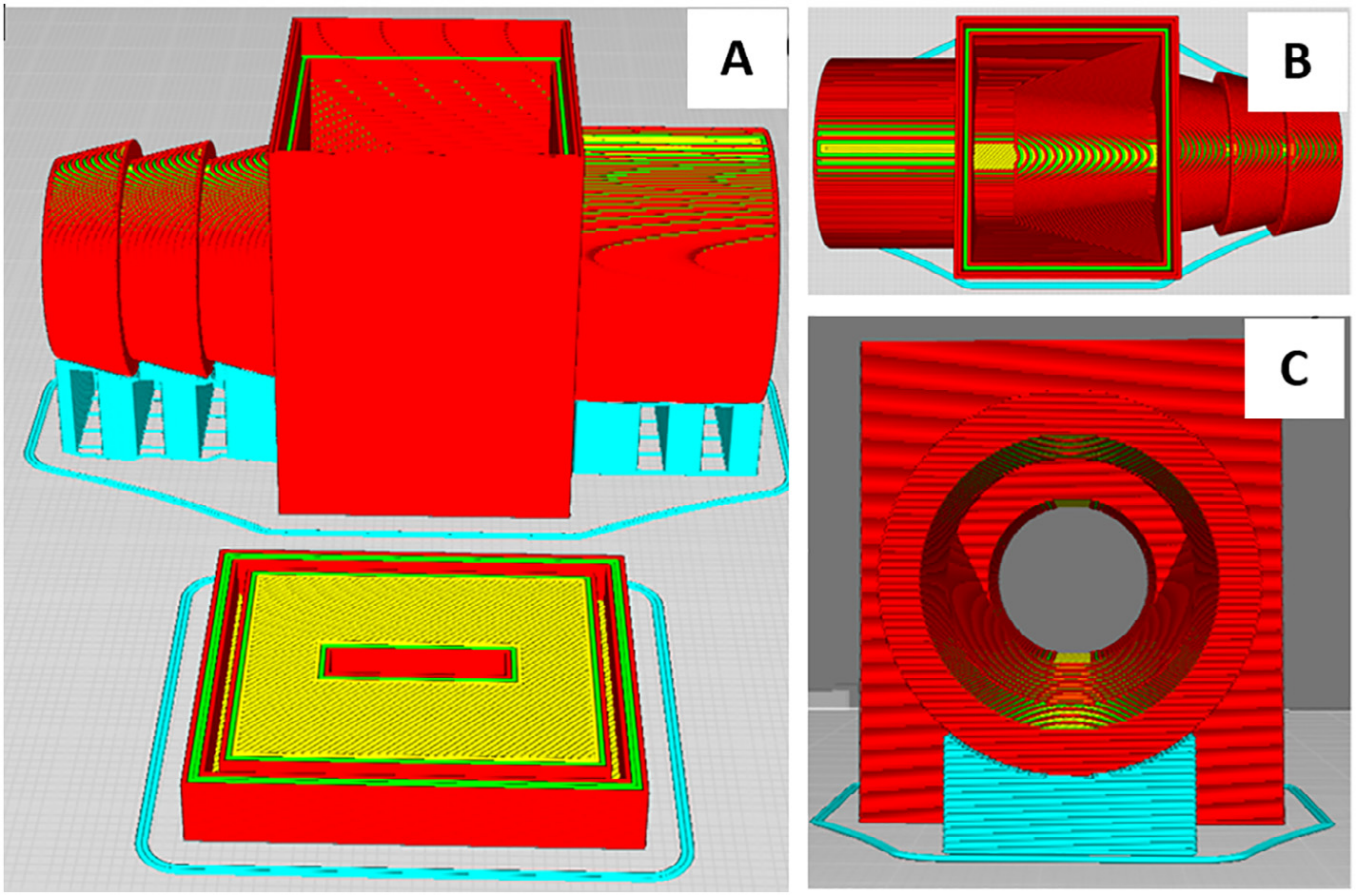
Junction box: Cura line types with support material in blue, shell in red, top/bottom in yellow, and inner wall in green. Overview of both the junction box and plate (A), top down view with a barbed input and a straight output for different tube diameters (B), side view down the output (C).

**Fig. 14:**
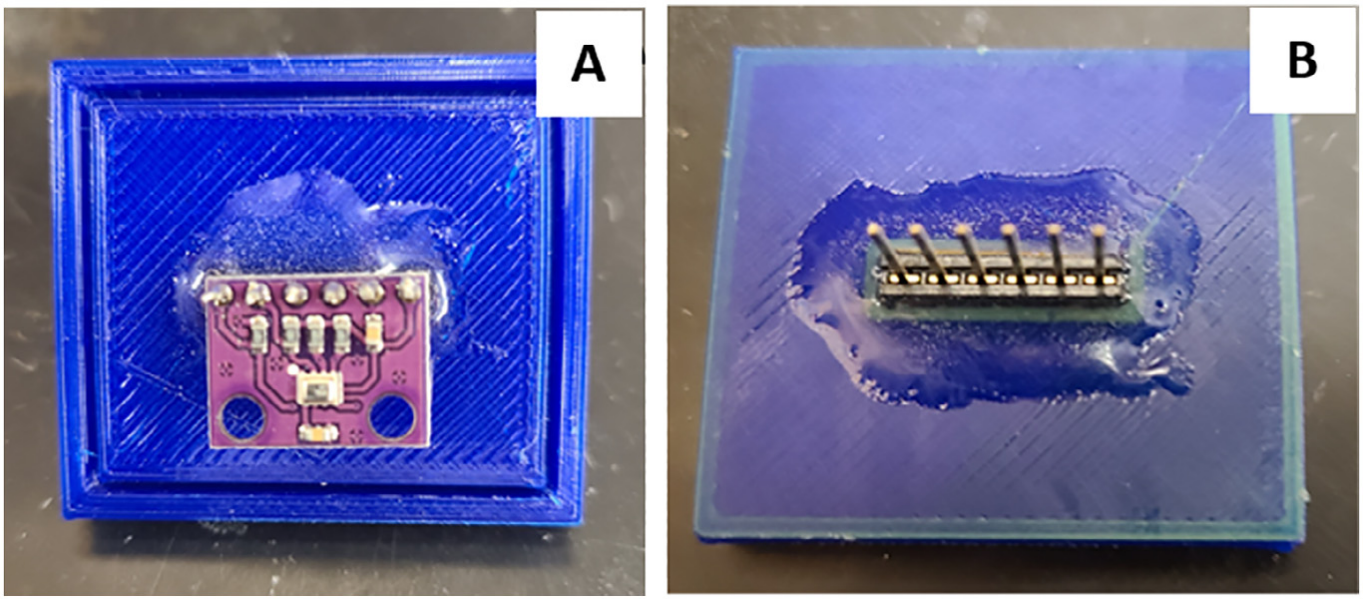
Pressure sensor cover: pressure sensor location (A) on bottom of junction box plate and epoxied 6 prong wire connection (B) on top of junction box plate.

**Fig. 15:**
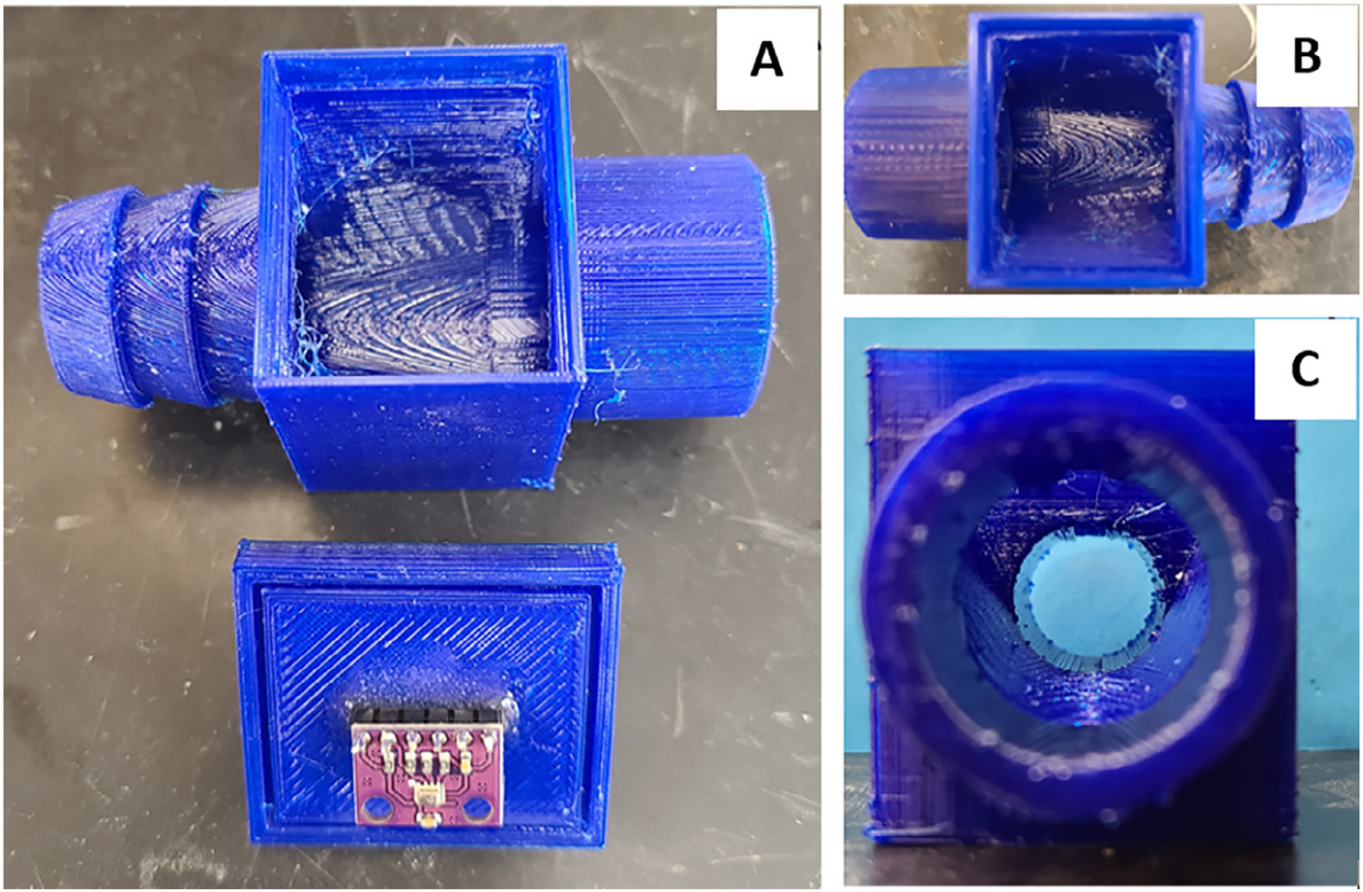
Junction box assembly: overview of printed junction box with press-fit plate and epoxied 6 prong attachment for pressure sensor wiring (A), top down view with a barbed input and a straight output for different tube diameters (B), side view down the output (C).

1. Obtain Ninjaflex filament

2. Obtain a RepRap-class 3-D printer capable of printing flexible filament

3. If it is necessary to alter the design Download the Junction_box_generator.scad from https://osf.io/fjdwz/ and download OpenSCAD from https://www.openscad.org/

4. Alter the input and output parameters in the design using the variable list as desired to fit the tubing sizes available

5. If there is no need to alter the design download the Pressure_sensor_junction_box_plate.stl and Pressure_sensor_junction_box.stl from https://osf.io/fjdwz/ and import into Cura or other open source slicer.

6. Download the material file from https://osf.io/fjdwz/ and import into Cura and set the appropriate print parameters individually or by importing the associated material file from https://osf.io/fjdwz/. 5 h and 36 min are needed to print the 39 g out of Ninjaflex at 50% infill, at 30 mm/s as determined by Cura (Fig. 13).

7. Using epoxy mount a 6-prong attachment for connection to the pressure sensor as seen in Fig. 13, then install the pressure sensor (Fig. 14). Fig. 15 shows the completed printed junction box assembly ready for use.

**Fig. 16:**
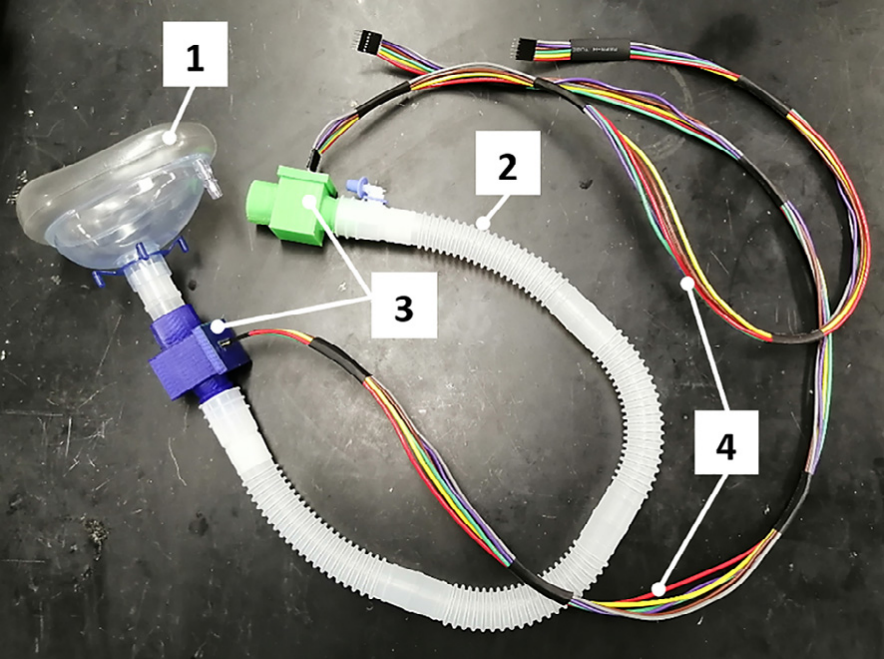
Breathing system assembly: 1) breathing mask, 2) 22 mm air tube, 3) junction boxes with pressure sensors inside, 4) cables for connecting pressure sensors.

**Fig. 17:**
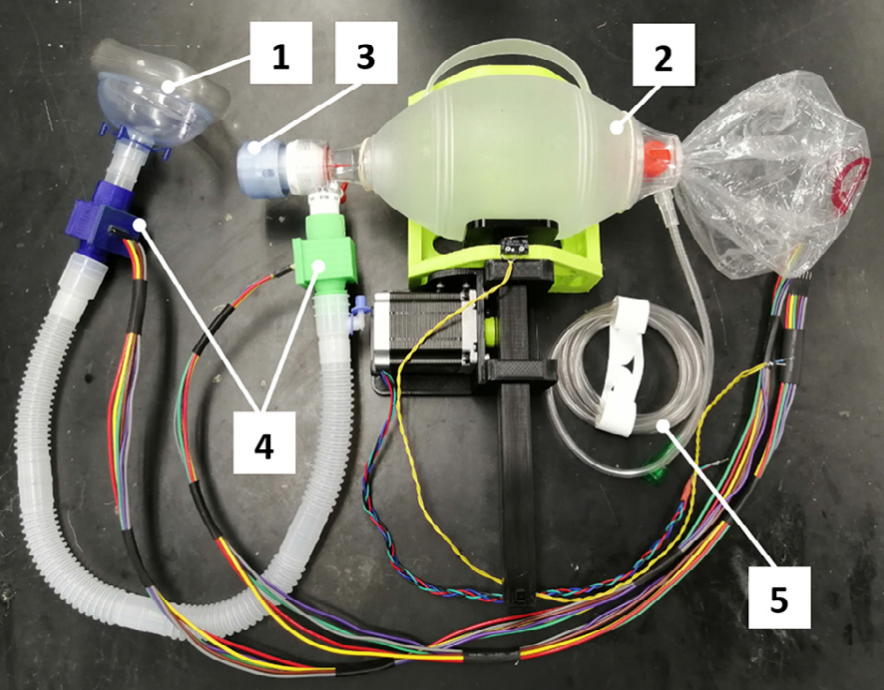
Breathing system connections: 1) breathing mask, 2) self-inflating bag, 3) PEEP valve, 4) junction boxes with pressure sensors inside, 5) oxygen hose (not involved in this work).

8 Connect cables and, junction boxes and tubes as shown on Fig. 16. Connect tubes to the BVM-bag (Fig. 17).

### 6.3 Control system assembly

**Fig. 18:**
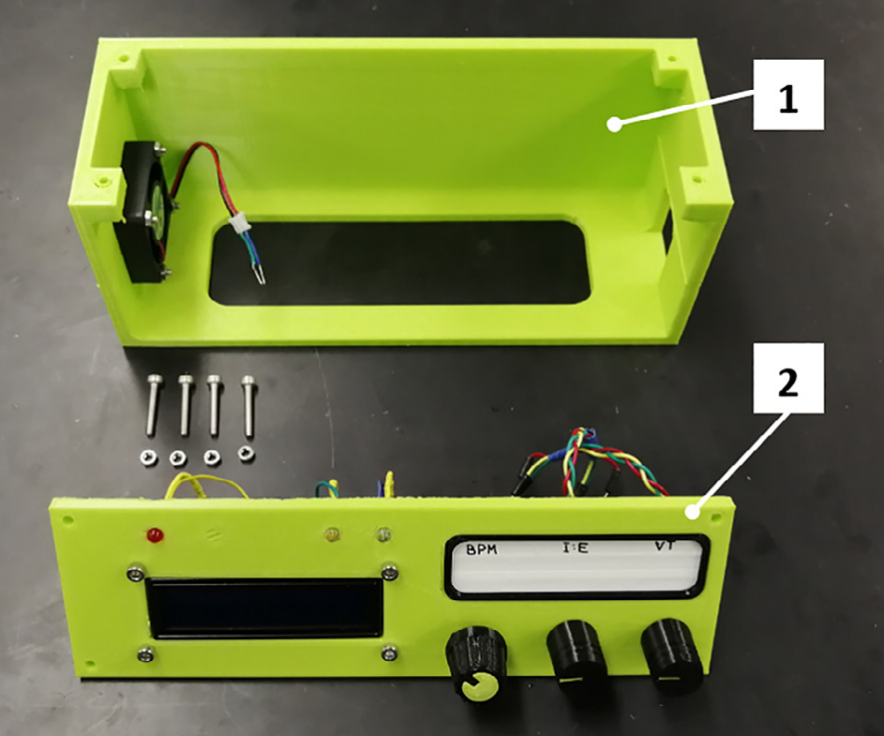
Assembly of the control system box: 1) base, 2) cover.

9 Print and assemble the case for the control system according to Fig. 18.

**Fig. 19:**
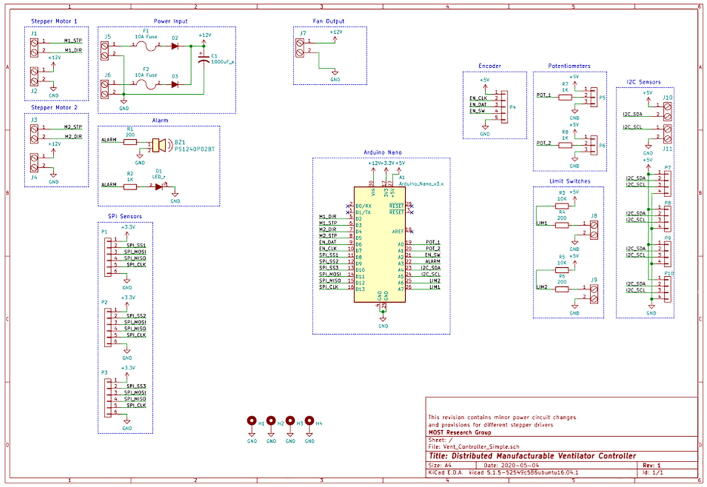
Electrical schematics of the control system.

**Fig. 20:**
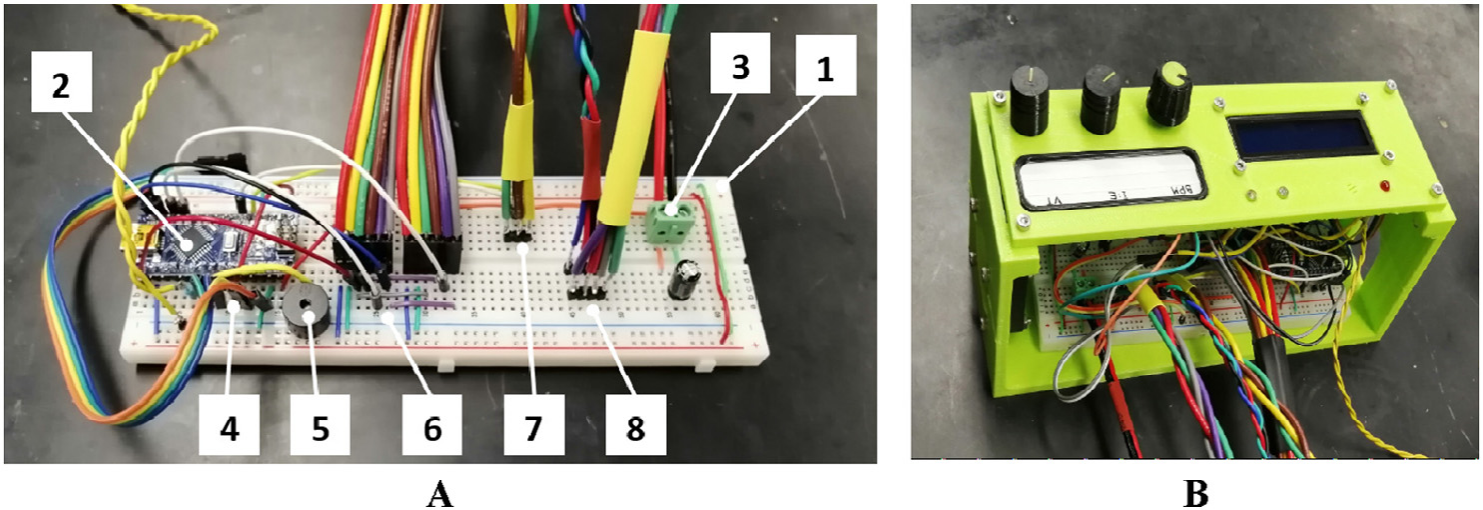
Breadboard assembly**:** A) breadboard with the components, B) breadboard installed inside the case, 1) breadboard, 2) Arduino Nano, 3) power supply connection, 4) LCD display connection via I_2_C bus, 5) alarm buzzer, 6) pressure sensors connection via the serial peripheral interface (SPI), 7) stepper motor control, and 8) stepper motor coils.

10. Connect the components according to the schematics (Fig. 19) and build a breadboard (Fig. 20, A). Note that the fuses in the schematic cannot be installed into the breadboard and must be omitted. Additionally, there are some sets of redundant or extra connections that are not needed for this specific implementation. Install the breadboard into the case (Fig. 20, B).

**Fig. 21:**
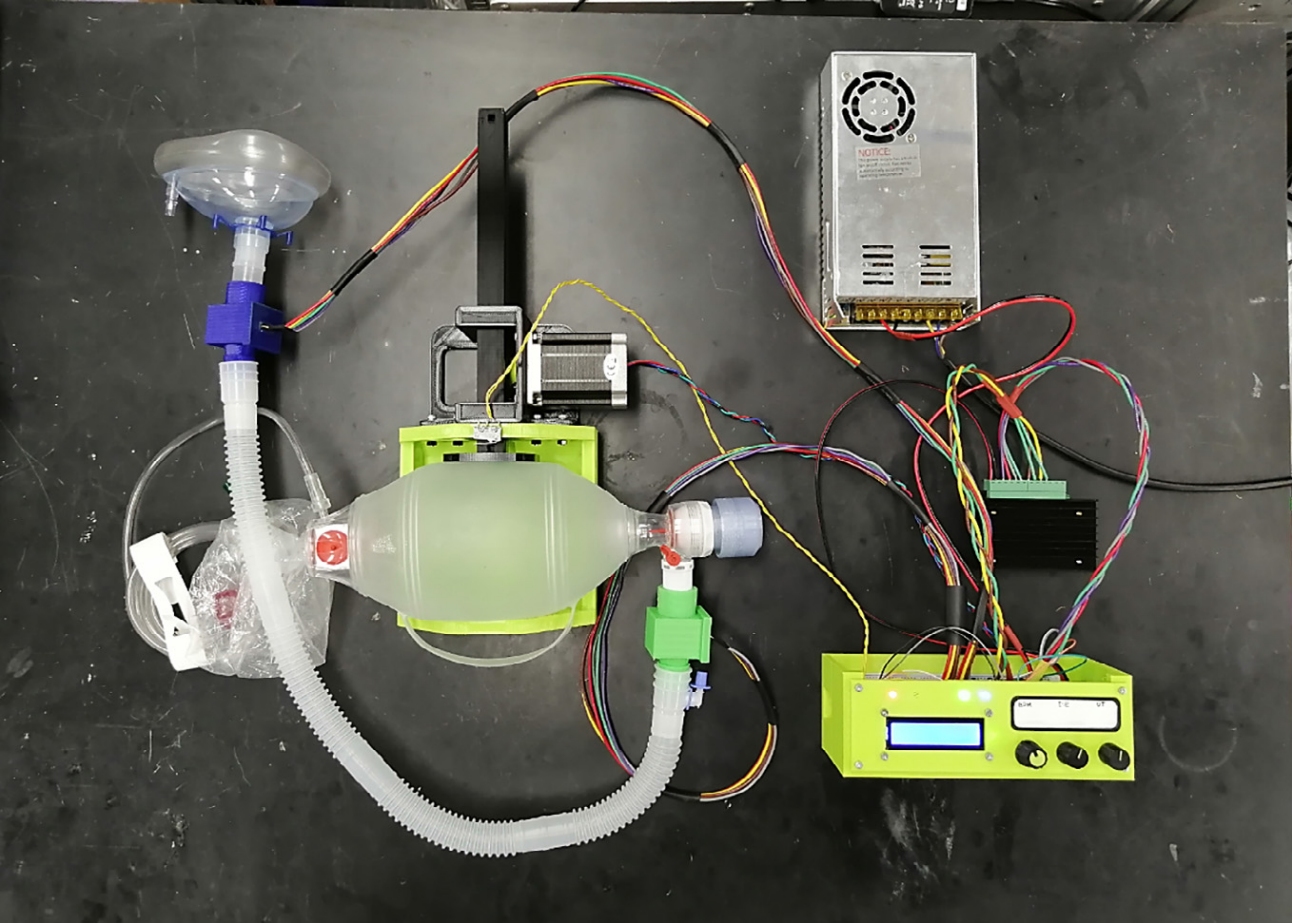
Complete assembly of the standalone automated BVM-based resuscitation system.

The complete system is then assembled as shown in Fig. 21.

10 Install the firmware by uploading the “arduino_firmware.ino” file to the Arduino Nano controller via Arduino IDE (https://www.arduino.cc/en/main/software).

## 7 Operation instructions

**Fig. 22:**
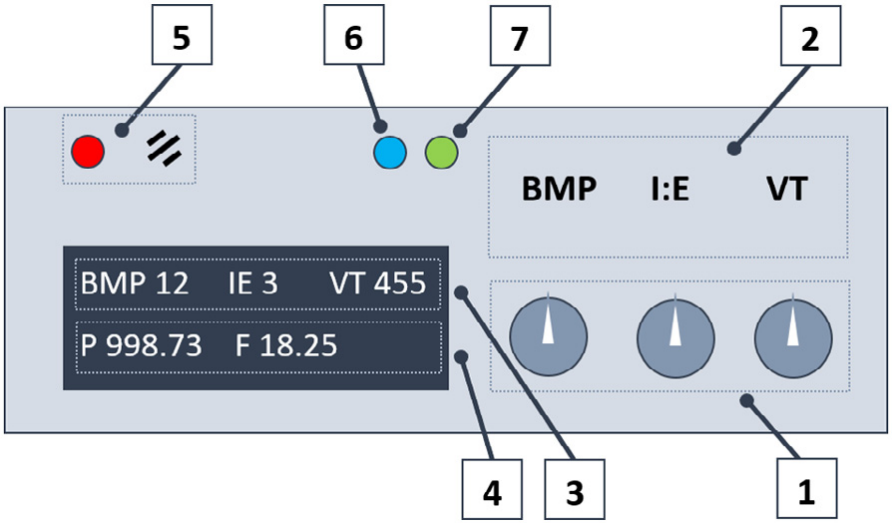
Control panel: 1) control knobs for breath rate, I:E ratio, and tidal volume, 2) text field, 3) input parameters displayed on LCD display, 4) feedback parameters (airway pressure and airflow), 5) alarm section (LED and buzzer), 6) inspiration indicator (motor pushes the self-inflated bag), 7) motor step command indicator.

Using the control knobs on the top panel (Fig. 22, component 1), a user must set the desired breathing mode and connect the patient to the mask.

The LCD (Fig. 22, components 3 and 4) displays the input parameters (V_T_, BPM, and I/E) and feedback (proximal airway pressure and estimated airflow). LEDs 6 and 7 (Fig. 22) reflect the motor operation mode. LED 5 signals an alarm when the proximal airway pressure exceeds the permissible range.

When using the device, there may be a danger of electric shock. Performed incorrectly, BVM-based ventilation can accelerate hypoxia and aggravate airway obstruction [116]. This can result in serious injury or death [117], [118], [119], [120], [121], [122], [123], [124], [125], [126]. According to the international “Medical Device Software” standard IEC 62,304 [127], [128], ventilators are class C medical equipment that can lead to patient death.

## 8 Validation and characterization

**Fig. 23:**
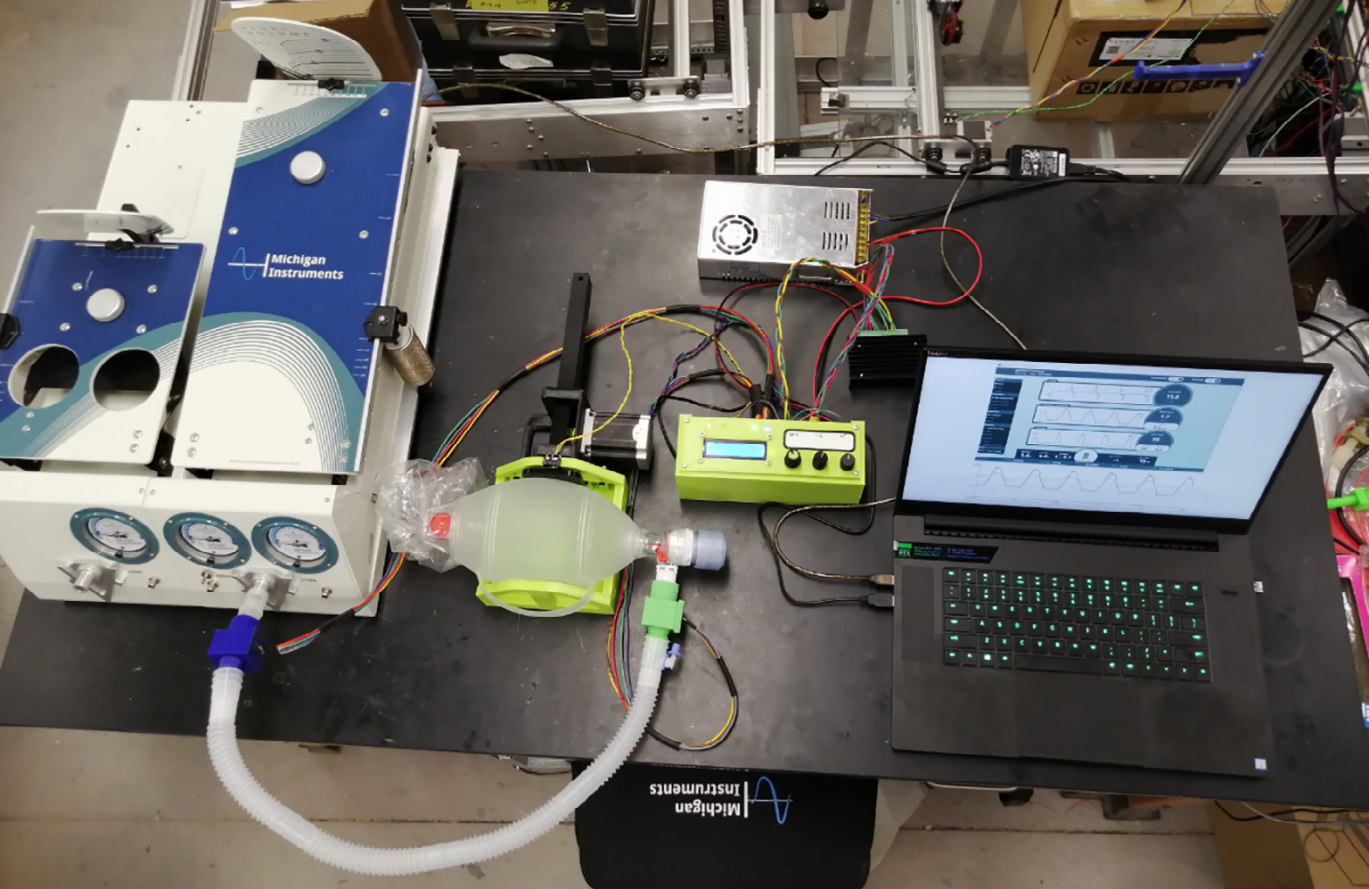
Testing procedure set up.

**Fig. 24:**
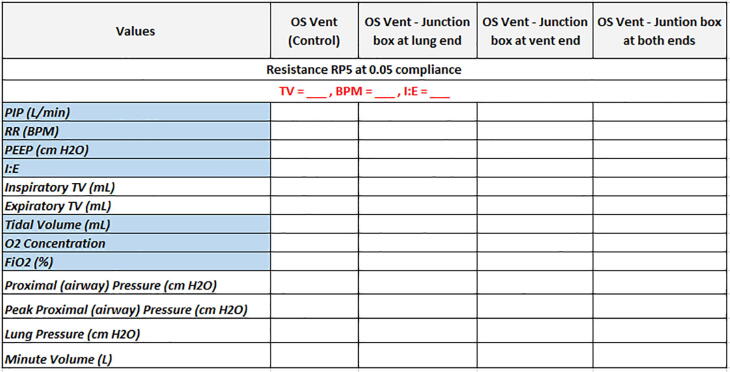
Example of the values collected for each test at a given tidal volume, respiratory rate, and an I:E ratio: values highlighted in blue represent the required metrics.

The mechanical design was experimentally tested for consistency, accuracy, and reliability using a Michigan Instruments Lung Simulator [106] as shown in Fig. 23. Table spreadsheets were created that compared values from outputs from Michigan Instruments Test and Training Lung software, PneuView3, [129] with target values. These variables included, peak inspiratory pressure (PIP), respiratory rate (RR), positive end-expiratory pressure (PEEP), I:E ratio, and tidal volume. A sample spreadsheet used for tests is illustrated on Fig. 24. The spreadsheets are included in the OSF repository.

Each experimental test was conducted at a set tidal volume (starting from 100, increasing to 800 at an interval of 100), a set respiratory rate (starting at 5 BPM, increasing to 15 at an interval of 5), and a set I:E ratio (1:2). The airway resistance was kept at a constant Rp5 [106] with a compliance of 0.05 to simulate a healthy adult lung. The PEEP valve was not touched to determine if it was consistent for all tests.

**Fig. 25:**
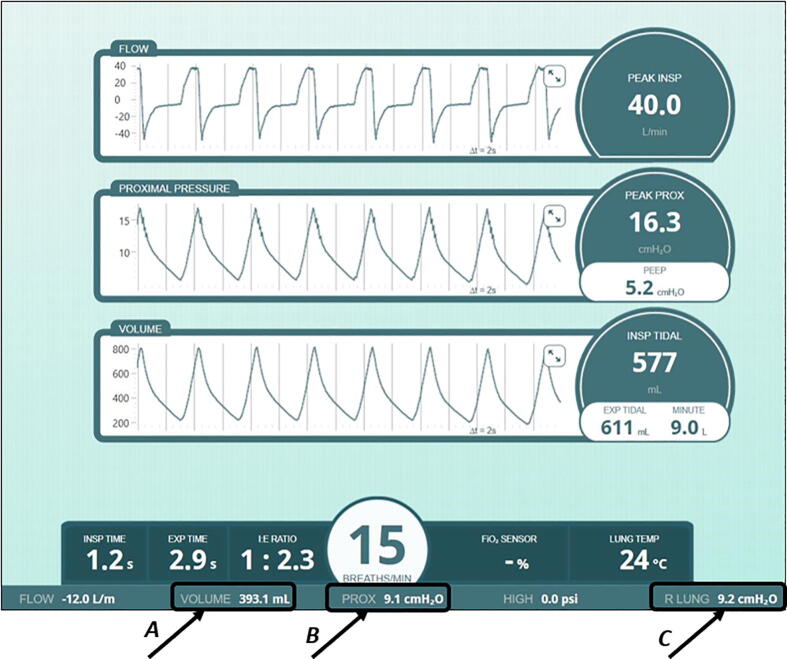
PneuView software, values recorded in real time: A) tidal volume. B) proximal (airway) pressure, and C) lung pressure.

The values for every measurement, excluding the flow, oxygen concentration, and FiO_2_ percentage, were recorded through the PneuView3 (Fig. 25) by taking a screenshot of the software screen once it became constant. While waiting for the data to become constant, the maximum values for tidal volume, proximal pressure, and lung pressure were recorded in real time.

Due to the data being recorded in real time, the values fluctuated as the tests went on. Statistical analysis was completed by calculating the standard error between each test using the built in STDEV.P function in Excel. Since the tests were run for three trials (N = 3) to determine repeatability, the standard error (SE) was found by the equation (7). This was completed for the most important values such as PIP, PEEP, tidal volume, proximal pressure, and lung pressure.

(7) $$\mathrm{SE}=\frac{\sigma }{\sqrt{N}}$$

where σ is the standard deviation of the parameter distribution and N is the number of observations.

A few changes were made between trials to gain more accurate data. This included attaching the rack pusher to the pinion, securing the valve bag with rubber bands, and switching out the gear used to push the rack forward. However, the data remained slightly inaccurate after the modifications. Future work should focus on designing a more stable mechanical set-up that will not need to be adjusted after a few hours.

Another metric that was analyzed by this protocol was to determine if adding a junction box was going to cause failures within the system, or if there was a specific location that the box should not be installed. The oxygen was not measured due to the ventilator using room air, thus, assuming that the O_2_ concentration and FiO_2_ percent were up to standards, it was also assumed that the hospital themselves would be able to observe these values using their technology and resources.

**Fig. 26:**
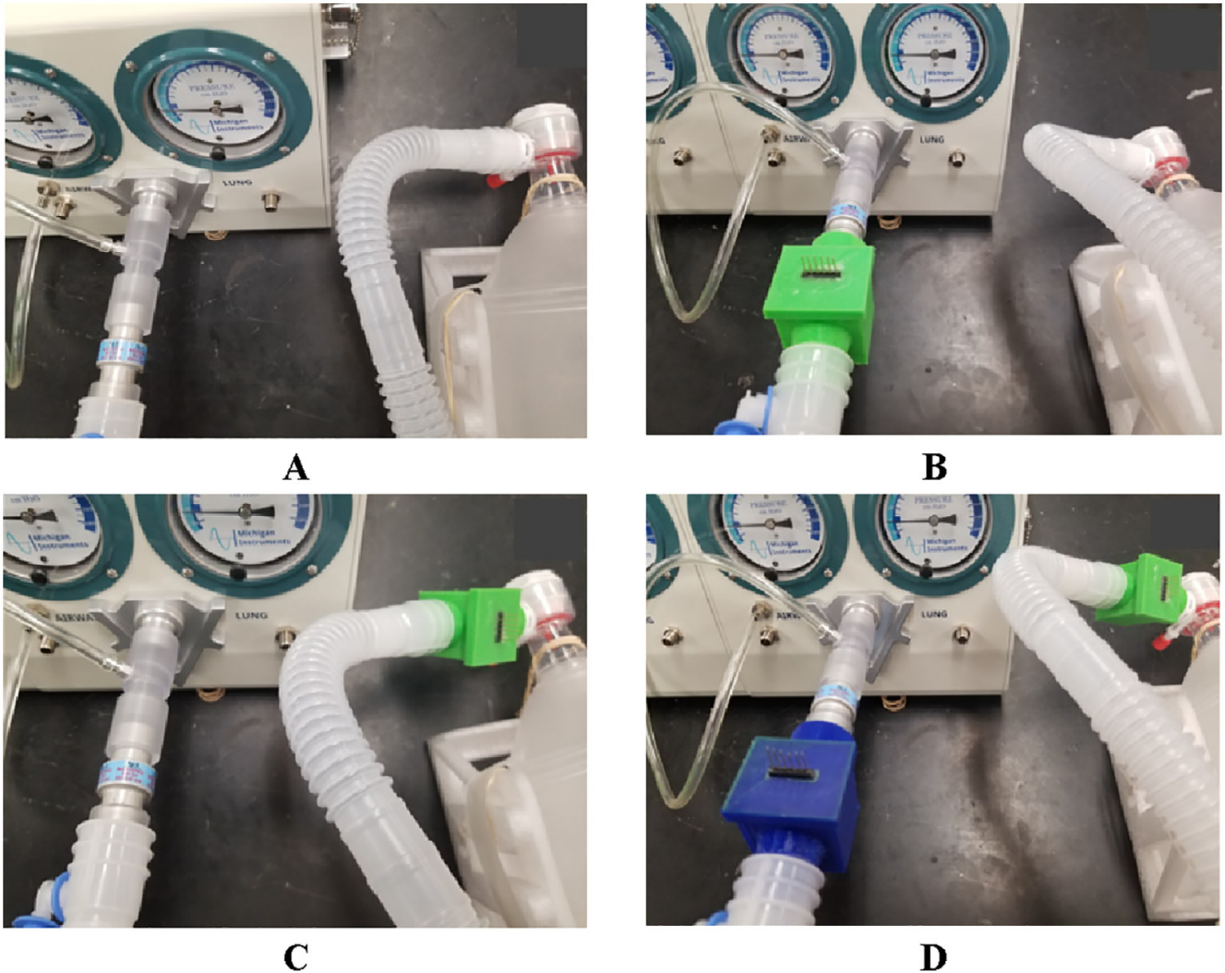
The placements of the junction boxes for testing: A) no junction box attached (control), B) junction box attached at the lung end of the connection tube, C) junction box connected at the ventilator end of the connection tube, and D) junction box connected at both ends of the connection tube.

There was a total of four tests that were completed using the previously mentioned protocols. These included tests where there was no junction box attached to the ventilator connection tube, with a junction box at each end of the tube separately, and finally with a junction box at both ends of the tube (Fig. 26). The wires connected to the pressure sensors were not used during the testing process. These were also completed a total of three time (N = 3).

**Fig. 27:**
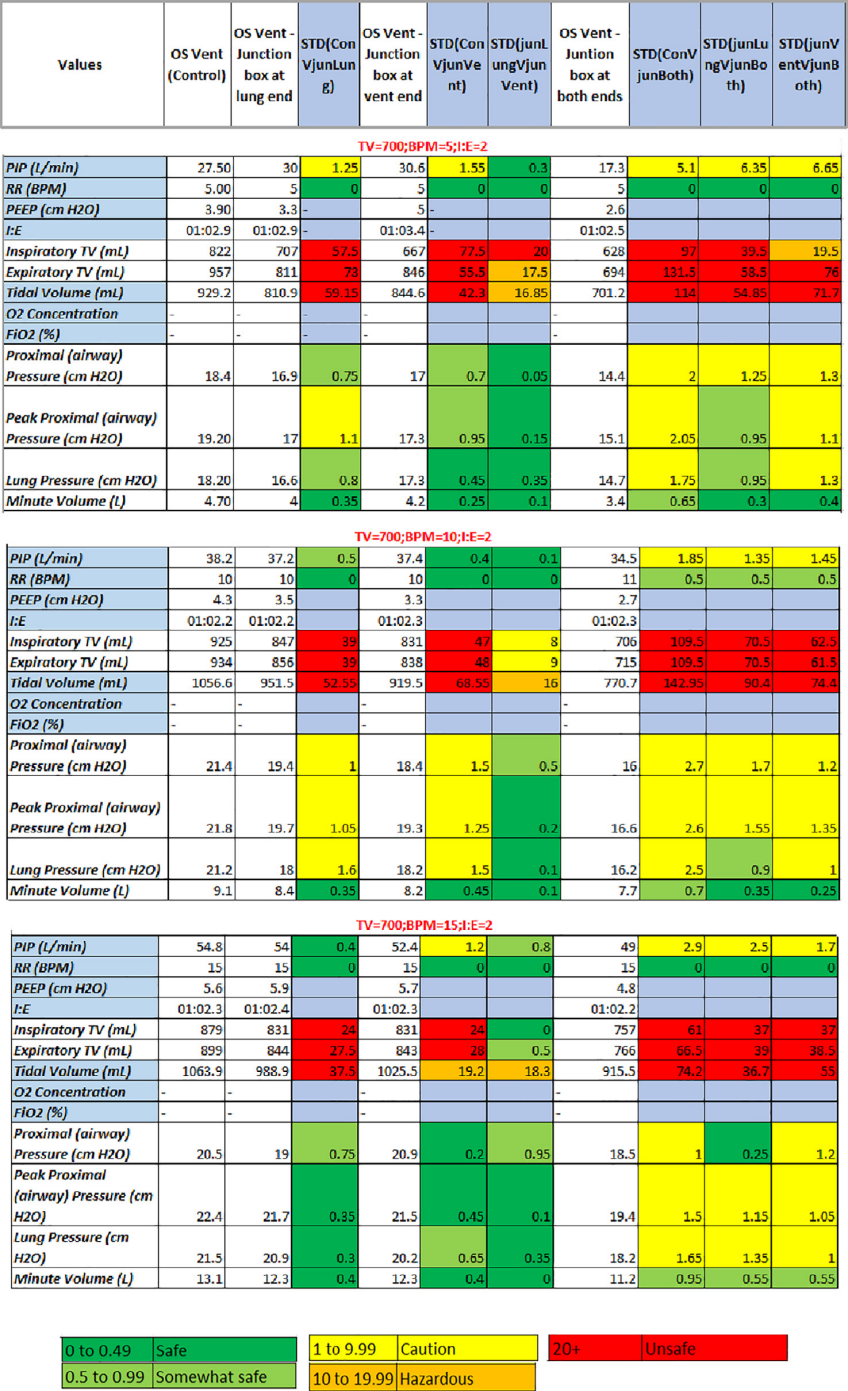
Representative data gathered for the junction box testing: set tidal volume of 700 mL, respiratory rate of 5, 10, and 15 BPM, and an I:E ratio of 1:2 for trial 1.

Both the green and blue boxes were epoxied between the pressure sensor and the lid. The meaningful difference being, the blue box did not contain super glue to hold the pressure sensor to the ports on the underside of the lid, whereas the green box did. After completing these tests, it was found that adding a junction box could cause significant changes in the data if the box was not assembled correctly. It was also seen that at low tidal volumes the lung was unable to calculate the majority of values, thus half the data could not be collected. In some instances the lung struggled to maintain consistent data causing values to be estimated. However, the main significant difference for each test, and trial, was the tidal volume. For the majority of tests, the tidal volume recorded was different than the volume manually set on the user interface. The standard deviations were also incredibly unsafe with the majority being over 60 mL for all three trials. A representative data set from trial 1 was created to show the similarities and differences between each test condition (Fig. 27).

The difference in tidal volume may simple be due to the design of the valve bag, undetected motor slippage, or the mechanical set-up of the ventilator. It can also be assumed that it was from the addition of junction boxes. In regard to having two boxes, the proximal and lung pressures were also not reliable. By looking, however, at the comparison between the single junction box tests, there was little to no standard deviation. Future work will delve into how to make the junction boxes more reliable, as well as how to maintain tidal volume.

Another investigation was based off the respiratory rate itself. It can be seen that the standard deviations between tests at 15 BPM are slightly lower than those of the 5 BPM test. This makes sense since there was a higher sample rate for calculations, and less time between breaths to let air escape through any leaks. The 10 BPM data could be a considered an outlier because the majority of values are above both the 5 and 15 BPM tests. This could have been caused by a shift in the valve bag, a slip of the motor, or inaccurate data gathered from the lung itself. The mechanical issues associated with this design should be addressed in future work to confidently confirm that the ventilator is consistent, accurate, and reliable.

**Fig. 28:**
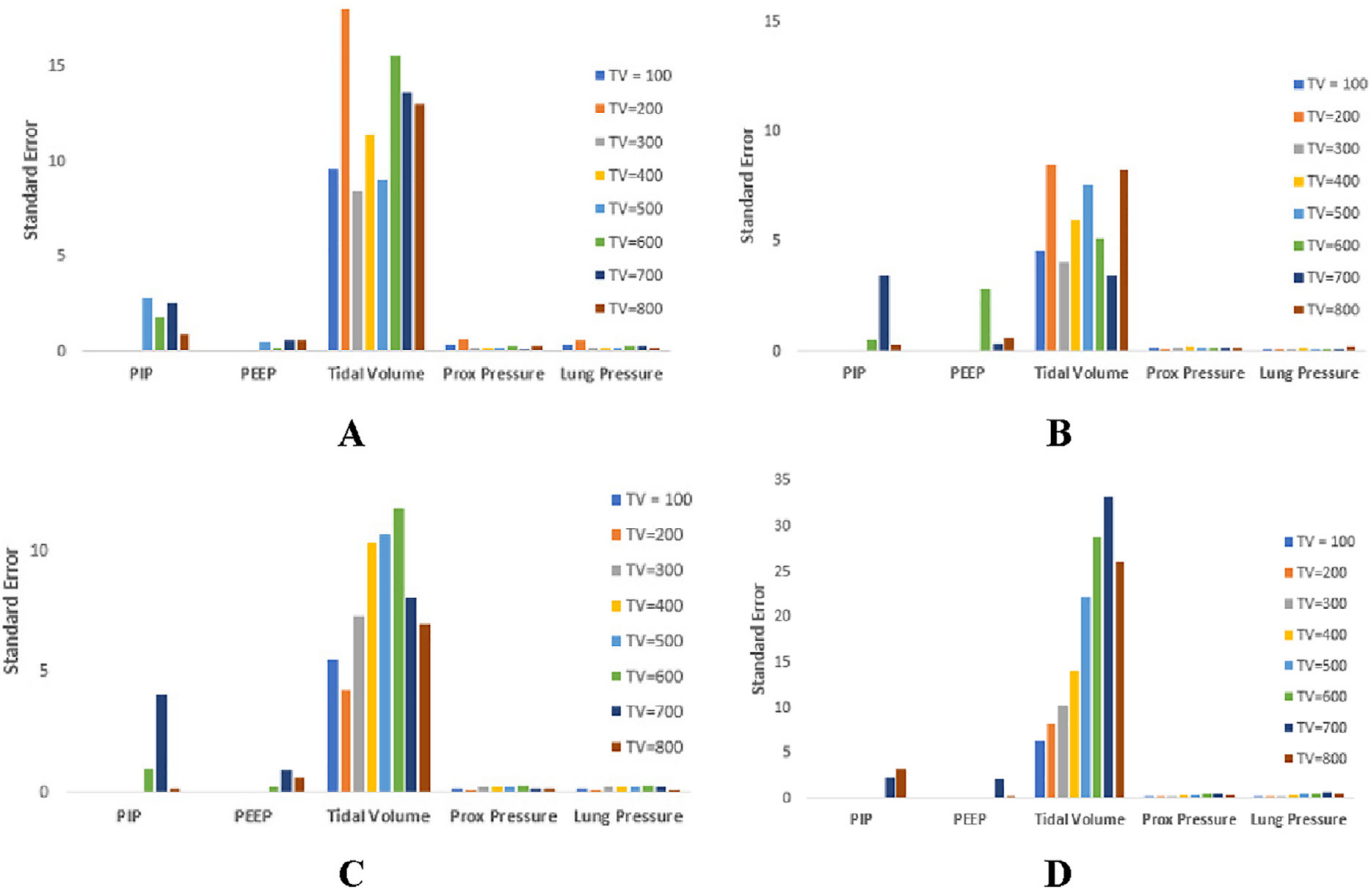
Representative standard error between each test for each position of junction box at a respiratory rate of 5 BPM: A) no junction boxes attached, B) junction box at lung end, C) junction box at ventilator end, D) junction box at both ends. Legend shows the tidal volumes that were input by the user.

The standard error results (Fig. 28) indicate, notably, significant difference between the means of the targeted tidal volumes.

This tidal volume was not what was manually input by the user, but the volume that was outputted by the PneuView software. It can be seen that for every tidal volume, and trial, the output TV experienced a high standard error. This is especially true with the addition of two junction boxes where the highest value was at a standard error of about 35 cm H_2_O, almost 20 cm H_2_O greater than the control trial. The PIP and PEEP values also showed inconsistency between trials however they were not as severe as the tidal volume values. The pressure metrics were relatively constant for each trial, with the exception of a few outliers. These outliers can be seen in the control trial at an input tidal volume of 200 cm H_2_O.

The open source ventilator here had alarms for 1) low pressure, 2) high pressure and 3) wire disconnect. Future work could consider adding oxygen concentration alarms, oxygen tube disconnection alarms, battery backup alarms and a mechanical failure alarm. The low/high pressure alarm was tested by manually squeezing, and releasing, the valve bag so that the pressure sensors detected pressures above 40 cm H_2_O, and below 5 cm H_2_O. The wire disconnection alarm was tested by manually unplugging the wires that were connected to the pressure sensors themselves.

The ventilator was then assessed on if it was able to pass the key ventilation specifications developed by the E-Vent Key Ventilation Specifications (v 27 March 2020) [74]. The first three tests were completed by adjusting the respiratory rate, tidal volume, and I:E ratio using the potentiometers installed on the circuit board from the minimum value to the maximum. The proximal pressure was limited by programming the pressure alarms to go off above 40 cm H_2_O. A plateau pressure limited to 30 cm H2O can be added by introducing an adjustable pause after the end of the inspiratory phase.

The PEEP values were confirmed by adjusting the PEEP valve connected to the exhale port of the valve bag. However, since the mechanical spring underlying the PEEP valve operation is a very sensitive part, even slight valve adjustment can lead to deviations of positive end expiratory pressure up to 4 cmH_2_O. The PEEP values depend on the tidal volume and respiratory rate. Thus, the PEEP could be stationary at one spot, but be different for a respiratory rate of 5 BPM than for a respiratory rate of 10 BPM. It should also be noted that various materials and printing parameters can lead to tolerance deviations, which makes the calibration of a mechanical PEEP valve a non-trivial task.

HEPA filters can be added in the future to determine the effect that a filter will have on the data. The e-Vent minimum requirements are met as shown in Table 2.

**Table 2:** Determination of meeting E-Vent Key Ventilation Technical Specifications [74].

Key Ventilation Specifications	E-Vent Recommendations	RepRapable OS Design
2.1. Control of Breathing Rate (breaths per minute)	8–30 BPM	5–45 BPM, controlled by user interface
2.2 Control of Tidal Volume (air volume pushed into lung in mL)	200–800 mL based on patient weight	100–846 mL, controlled by user interface
2.3 Control of I/E Ratio (inspiratory/expiration time ratio)	best if adjustable between range of 1:1–1:4	Adjustable between range of 1:1–1:4, controlled by user interface
2.4 Assist detection pressure. When a patient tries to inspire, they can cause a dip on the order of 1–5 cm H_2_O, with respect to PEEP pressure (not necessarily = atmospheric).	Required	Can be added based on pressure [132] or temperature [66] feedback
3. Airway pressure must be monitored	Required	Contains two pressure sensors connected between the ventilator and patient via an airway tube, pressures are shown on the LCD screen
3.1.1 Pressure limits: Max pressure	40 cm H_2_O	Alarms sound if pressure exceeds 40 cmH_2_O
3.1.2 Pressure limits: Plateau pressure	30 cm H_2_O	Can be added by introducing a pause at the end of the inspiratory phase [132]
3.2 Passive mechanical blow-off valve	40 cm H_2_O	Can be purchased together with the self-inflating bag kit
3.3.1 Monitor plateau pressure	Clinician viewable	Can be added by modifying the control algorithm
3.3.2 Monitor PEEP	Clinician viewable	Can be added, need software to see the quantitative value
3.4 PEEP	5–15 cm H_2_O	2–11 cm H_2_O based on observed data during testing
4. Manual clinician override	Failure of automatic ventilation allows conversion to immediate ventilation.	Yes
5. Ability to use ventilation on room air. Implemented with an oxygen/air gas blender that some hospitals already have.	Required for emergencies	Yes
6. HEPA filtration on the patient’s exhalation	Required because COVID-19 can be aerosolized	Can be added, HEPA filters can usually be purchased alongside manual resuscitator bags.
8. Failure conditions result in alarm	Required	Alarms sound if pressure exceeds the allowed limits from 5 to 40 cmH_2_O

Based on the trials the capabilities of the open source ventilator design include:

1. Maintaining a steady tidal volume, respiratory rate, and I:E ratio.
2. Containing multiple tidal volume values ranging from 100 mL to 846 mL at intervals of one.
3. Maintaining constant motor speed with no slippage below any tidal volume of 800 mL for a respiratory rate less than, or equal to, 15 BPM.
4. Creating consistent data graphs for flow, pressure, and volume.
5. Motors are able to quickly adjust to changes in tidal volume, respiratory rate, and I:E ratio.
6. Rack pusher increases total tidal volume and pressure that can be achieved.
7. All parts are 3-D printable on any RepRap-class printer excluding the electronics.
8. Parts can be easily changed in case of a failure.
9. Parts can be cleaned and sanitized.

## 9 Limitations and future work

The limitations of the final ventilator design include:

1. Incorporating the possibility of self-inflating bag displacement, as well as the accuracy of the pusher rod travel distance calibration, the tidal volume may differ from the set value within the standard error, which is approximately 35 mL.
2. During the tests, the NEMA-23 stepper motor was operated with the maximum current in the windings to cover the working range of the tidal volume and respiratory rate. These conditions lead to the excess heat buildup in the motor and the need for heat dissipation after several hours of continuous operation. Thus, to ensure ventilation modes with a tidal volume of more than 500 mL and a respiratory rate of more than 15 breaths per minute, it is necessary to use a motor cooling system in the form of a heat sink and/or active airflow.
3. It is recommended that spring washers be used in the motor mounting system to prevent possible bolt loosening due to motor vibrations.
4. During compression, the self-inflating bag may shift and rotate in the bag support, which will lead to a deviation of the set ventilation parameters. An elastic band is used as a fixing component, however, in the future, it is necessary to redesign the system to make bag movement physically impossible.
5. Valve bag is limited to the amount of airway pressure that can be achieved in a cycle.
6. Valve bag could be cause of shifting, and inconsistent, tidal volumes between tests.
7. Ventilator has to be taped down to a stable surface (i.e. piece of wood or clamp) to avoid vibrations that cause movement.
8. In the process of bag compression, there is a possibility of the pinion gear steps slippage, both due to insufficient motor torque and due to the fastening of the pinion to the motor shaft. In the future, this problem will need to be solved as follows:
  a. Add an extra screw securing the pinion to the motor shaft
  b. Provide software protection against slippage by returning the pushing rod to its “home” position (hitting the limit switch) every N number of steps.
  c. Implement an alarm signal in the event of the motor steps skipping (unexpected closure of the limit switch).
9. A large pusher can mechanically separate from the pushing rod due to the force exerted by the self-inflating bag at high tidal volumes. This problem can be solved by using a metal screw to secure the pusher.
10. It is hard to maintain a positive end-expiratory pressure control due to the difficulty of calibrating the PEEP valve. In the future, it is necessary to implement a software calibration procedure of the PEEP valve or to use a ready-made calibrated industrial design.
11. Junction boxes can cause leaks if not sealed correctly, reducing values drastically for low breathing rates.

In the future, the developed device can be improved by including the following modifications:

### 9.1 Electrical and software

**Fig. 29:**
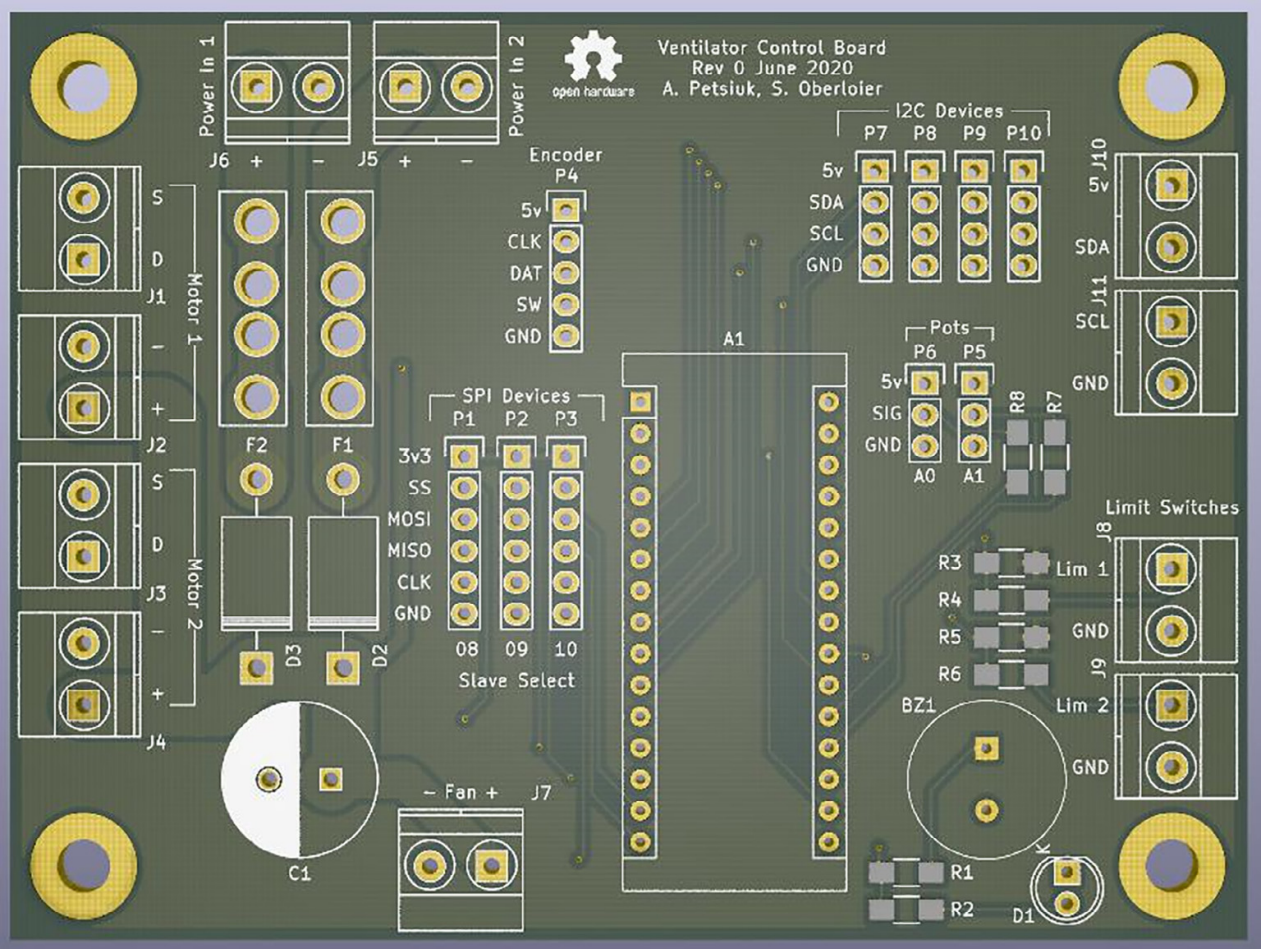
Printed circuit board design.

• Create an assistant mode based on feedback from the pressure sensors
• Add alarms such as “Power disconnect”, “Gear slippage”, and “Critical PEEP”
• Replace the breadboard with a printed circuit board (PCB) (Fig. 29), which is provided in the OSF repository. The implementation of a PCB will reduce the cost of the system, as the board will cost $2.37USD per unit. The PCB replaces the $7.90USD breadboard, while adding robustness, clear labeling and a more compact design.

### 9.2 Mechanical part

• Develop more efficient support for self-inflating bags
• Improve PEEP valve performance or replace it with a ready-made calibrated industrial part
• Add HEPA filter
• Add a mechanical pressure relief valve as the default option
• Add cooling system for the motor
• Work towards a more completely distributed-manufacturable device (e.g. replace all the current purchased components for those that can be manufactured on site from feedstock).

### 9.3 Medical functionality and testing capabilities

• Conduct longevity validation to ensure long-term reliability for multiple patients and determine the lifetime of the device
• Add FiO_2_ and O_2_ sensors
• Conduct medical validation with a clinician specialist to ensure the device is clinician friendly
• Complete sterilization and testing to ensure that the device can be cleaned using conventional methods and chemicals. The chemical compatibility of commercial 3-D printing materials is well known [84] and this provides several chemical sterilization pathways that would need to be tested.

Finally, it should be noted that this device was designed for distributed manufacturing, which is currently discouraged by the nature of some regulations (e.g. the FDA certifies a device and a manufacturer as one). This device is not yet approved for clinical use. Future work is needed to develop integrated testing facilities for the open source ventilator to enable rapid manufacturer certification as well as full regulatory approval of the device. This will involve meeting medical device standards such as ISO 80601-2-12:2020 - Medical electrical equipment, ISO 5367:2014 - Anaesthetic and respiratory equipment, and IEC 62304:2006 - Medical device software.

## 10 Conclusions

The comparative characteristics of modern ventilators under development [113], [130], as well as the medical recommendations of experienced anesthesiologists [131], allow determining the main advantages and disadvantages of the developed system. Ventilators created by developers around the world can be divided into two main groups: 1) ventilators based on self-inflating bags [132], [69], [133], [134], [135], [136], [137], [138], and 2) ventilators based on compressors and pumps [139], [140], [141], [142], [143]. The main drawback of most existing projects is that the main stages of the design process, such as calculating of the required motor power, developing a mechanical compression system, feedback signal processing algorithms (pressure, temperature, flow, etc.), developing a cooling system based on temperature parameters of motors, are not well documented. Ventilators based on pumps often have advanced functionality that allows preparing gas mixtures, moisturizing the circulated atmosphere, and providing an autonomous assistance mode. The main disadvantage of such systems is the complexity of manufacturing, expensive and sometimes inaccessible components, as well as the difficulty in configuring and calibrating, which requires considerable expertise and experience from the user. BVM-based ventilators are easy to replicate and consist of low-cost, readily available components. The advantage of these systems is the ability to release a clinical specialist for a certain period of time to work with other patients. Such an automated apparatus significantly surpasses manual compression in accuracy and stability. Some of the considered BVM-based models, however, have a complex design with expensive components (personal computer, programmable logic controller, etc.) that may demand complex software algorithms. Many of these projects also did not put enough stress on testing.

In order to compare the development of open source ventilators, a five-point validation system has been developed for all types of ventilators, based on criteria such as openness, buildability, community support, functionality, reliability, COVID-19 suitability, clinician amiability [144]. Based on applying this metric the following can be concluded about the developed system described in this study:

• Fully open source and well-documented
• Easily reproducible
• Has been tested for pressure and volume limits with respiratory rate and tidal volume control
• Has critical emergency alarms
• Consists of standard components and connection blocks

Although the developed ventilation system is inferior to certified medical ventilators in the number of available modes, the open source device is far less costly and is able to be deployed by means of distributed manufacturing. In addition, the open source ventilator described and tested here surpasses the capabilities of manual BVM-based ventilation in the accuracy of reproducing predetermined breathing modes, as well as in the stability of the repetition of respiratory cycles. Future work is necessary to further develop the system tested in this work for acceptable deployment in clinical environments, however, the nature of the design is such that desired features are relatively easy to add and test using protocols and parametric design files provided by this study.

## Declaration of Competing Interest

The authors declare that they have no known competing financial interests or personal relationships that could have appeared to influence the work reported in this paper.
